# An Injectable, Self‐Expanding Hemostatic Foam for Field‐Compatible Postpartum Hemorrhage Management

**DOI:** 10.1002/advs.77669

**Published:** 2026-09-27

**Authors:** Charlotte Meyer, Noël Strasser, Ilakkiya Ravindrarajah, Elsa Wrenger, Kristian Ivkovic, Jan Ševčík, Sima Šarčević, Lenka Červenková, Jáchym Rosendorf, Václav Liška, Alexandre H. Anthis, Thomas Rduch, Inge K. Herrmann

**Affiliations:** ^1^ The Ingenuity Lab Balgrist University Hospital Zurich Switzerland; ^2^ Department of Mechanical and Process Engineering (D‐MAVT) Nanoparticle Systems Engineering Laboratory Institute of Energy and Process Engineering (IEPE) ETH Zurich Zurich Switzerland; ^3^ Department of Materials Meet Life Nanomaterials in Health Laboratory Swiss Federal Laboratories For Materials Science and Technology (Empa) St Gallen Switzerland; ^4^ Faculty of Medicine University of Zurich Zurich Switzerland; ^5^ Department of Surgery Faculty of Medicine in Pilsen Charles University Prague Czech Republic; ^6^ Biomedical Center Faculty of Medicine in Pilsen Charles University Prague Czech Republic; ^7^ Department of Gynecology Cantonal Hospital St Gallen (KSSG) St Gallen Switzerland

**Keywords:** alginate, chitosan, drug delivery, global health, postpartum haemorrhage, tamponade, tranexamic acid

## Abstract

Postpartum hemorrhage (PPH) remains the leading cause of maternal mortality worldwide and a major contributor to global morbidity. Although several interventions exist in high‐income countries, current solutions often suffer from poor field‐compatibility, limited efficacy, complex application, insufficient hemostatic support, and difficult removal. Here, a hemostatic foam designed for field compatibility and made from stable precursors, together with an easy‐to‐use delivery device for treating life‐threatening PPH is introduced. The device enables rapid, user‐friendly generation and intrauterine application of a hydrogel foam, which controls bleeding through both coagulation support and tamponade pressure before safely degrading. The expanding foam achieves the target pressure range of standard intrauterine tamponade (67–92 mmHg), confirmed across in vitro, ex vivo, and in vivo models. Induced degradation produces 76 ± 8% mass loss within 6 h, with storage modulus dropping from 2.98 ± 0.08 kPa after fabrication to 1.60 ± 0.25 kPa after partial degradation. Incorporation and controlled release of tranexamic acid and cefuroxime show versatility in topical drug delivery. Beyond extensive ex vivo testing, rapid, controlled deployment in vivo in a swine model is demonstrated. By integrating mechanical and chemical mechanisms, this injectable foam provides effective bleeding control, with a design well suited to low‐resource settings.

## Graphical abstract

Postpartum hemorrhage is the leading cause of maternal death worldwide, yet many treatments are hard to use outside well‐equipped hospitals. This work introduces an easy‐to‐use device that rapidly delivers a degradable foam into the uterus. The foam stops bleeding through pressure and clotting support, offering a practical option for low‐resource settings where it is needed most.

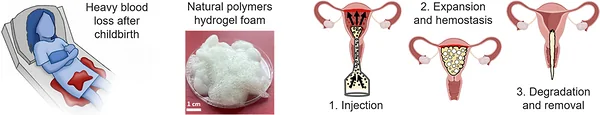

## 1 Introduction

Despite major advancements in modern medicine, hemorrhage remains a leading cause of morbidity and mortality worldwide. This is particularly critical in maternal health, where inadequate control of bleeding can lead to life‐threatening complications, lasting physical and psychological consequences, and a disproportionate burden on low‐ and middle‐income countries, where access to adequate medical resources is limited. Postpartum hemorrhage (PPH) is one of the leading hemorrhagic complications with a substantial impact on maternal health. PPH is defined as a blood loss of more than 500 mL from the genital tract after childbirth [1]. PPH affects up to 5% of births, causing around 20% of maternal deaths annually, with 99% occurring in low‐ and middle‐income countries [1, 2]. The 2023 WHO roadmap highlights PPH as a major maternal health challenge [1], driving innovations like cost‐effective diagnostic tools to measure blood loss [3]. Advancing PPH research supports progress toward SDG 3.1, aiming to reduce maternal mortality to fewer than 70 per 100 000 live births [4]. In 90% of cases, PPH is caused by uterine atony, where the uterus muscles are unable to contract and close the spiral arteries left open by the placenta expulsion after delivery [5].

The first‐line treatment of PPH is the intravenous injection of uterotonics, such as oxytocin or prostaglandins [6]. If unsuccessful, other conservative treatments are applied, such as intrauterine devices [7]. Among them, balloon tamponade is mostly used and is based on inflating a balloon catheter inside of the uterus to apply pressure on the uterine walls. Still, this technology is prone to dislocation of the balloon, infections, and poor conformation to the uterine cavity [8, 9]. Additionally, the application procedure is time‐consuming and requires highly skilled personal and appropriate clinical settings. Like other purely mechanical approaches to hemostasis, the balloon tamponade shows limited efficiency in case of a hindered coagulation system [10]. Hemostatic materials such as chitosan gauze packing (Celox PPH) offer an alternative to balloon tamponade, especially in resource‐limited settings [11]. Despite high success rate in PPH management, the new WHO guidelines do not recommend the use of chitosan gauze packing [12]. The failure risk of chitosan gauze treatment increases in case of placenta previa and severe hemorrhage [13]. In addition, chitosan residues might stay in the uterine cavity [14], which can cause adverse effects, including inflammations and infections. Taken together, current solutions for the treatment of PPH are unsatisfactory because of difficulties with application, biocompatibility, and removal.

A myriad of hemostatic materials taking different shapes such as hydrogels, sponges, foams, or powders have been investigated and developed for traumatic bleeding [15]. While most of the research focuses on injuries of organs such as the liver [16], the skin [17], or the kidneys [18], studies on gynecological bleeding modelling and management remain underrepresented. Recent work by Miller et al. has explored an injectable and adhesive hydrogel with hemostatic activity for PPH management [19]. They demonstrated reduced clotting time and effective bleeding control in benchtop and rat models. However, the authors reported low burst strength (∼26–30 mmHg) and mentioned the need for an additional adjunct to manage the pressurized flow of PPH. The authors further noted that catheter‐based delivery is limited by the need to localize bleeding sites, which is often impractical in PPH. A few new devices showing promising results have been designed for PPH treatment, such as an intrauterine vacuum device (Jada System) [20]. However, its use is constrained by the need for reliable vacuum suction, high cost, a cervical dilation of more than 3 cm, and limited suitability for PPH caused by placenta accreta spectrum disorders [7]. Existing hemostatic materials have also been repurposed for PPH. For example, XStat mini‐sponges, originally designed for incompressible hemorrhage, have been adapted for uterine use with a newly developed applicator that encloses the sponges in a removable net [21]. While effective in their primary indication, this adaptation remains limited by an unsatisfactory removal method, lack of biodegradation, and reduced efficacy in coagulopathic patients due to its purely mechanical hemostatic mechanism. PPH was also previously explored as an indication for ResQFoam, a polyurethane foam injection device designed to achieve hemostasis by exerting pressure on the uterine walls through foam expansion [22]. However, the technology was later redirected toward managing traumatic abdominal hemorrhage as a pre‐surgical containment strategy [23]. Moreover, ResQFoam is hindered by concerns regarding markedly elevated intra‑abdominal pressure, uncontrolled compression of intra‑abdominal organs and vessels, and the requirement for surgical removal [24]. Taken together, these approaches share limitations. Mechanical devices generate tamponade at clinically relevant pressures but conform poorly to the uterine cavity, do not contribute to coagulation, and lose efficacy when the clotting capacity is impaired [8, 10]. Injectable hydrogels combine both functions, yet reported burst strengths remain well below the pressures encountered in PPH and delivery depends on localizing individual bleeding sites [19]. Expanding foams reach the required pressures but without control over the resulting compression [25]. Finally, none of these technologies offers removal that avoids surgery or manual retrieval. An effective PPH treatment must therefore fill and conform to the irregular uterine cavity without site‐specific targeting, generate sustained pressure within a controlled and clinically safe range, actively support coagulation independently of the patient's own clotting capacity, and be removable on demand while remaining deployable outside a well‐equipped clinical setting.

Here, we present an in situ‐forming injectable hydrogel foam designed for rapid field deployment and controlled degradation as a novel treatment for PPH. The foam comprises alginate and chitosan and is generated from a field‐reconstitutable precursor solution using a custom cream‐whipper device. Hemostasis is achieved through a dual mechanism: mechanical tamponade via foam expansion and chemical support of coagulation. We demonstrate sustained pressure generation across in vitro, ex vivo, and in vivo models, including a swine model using a newly developed minimally invasive intrauterine pressure‐monitoring method. Coagulation enhancement is validated in vitro on fresh human blood as well as a human placenta, and in vivo in a porcine liver hemorrhage model. The foam is engineered to undergo stimuli‐responsive degradation, ensuring safe and timely removal. The system therefore addresses the limitations outlined above within a single device by providing cavity‐filling deployment without bleeding‐site localization, sustained pressure generation in a controlled range, procoagulant activity independent of the patient's clotting capacity, and stimulus‐triggered removal avoiding surgery or manual extraction. Its field compatibility, simplicity, biocompatibility, and low cost position it as a promising, accessible alternative for resource‐limited settings, where the scarcity of effective PPH treatments continues to endanger maternal health.

## 2 Results and Discussion

### 2.1 Hydrogel Foam Composition and Delivery

**FIGURE 1:**
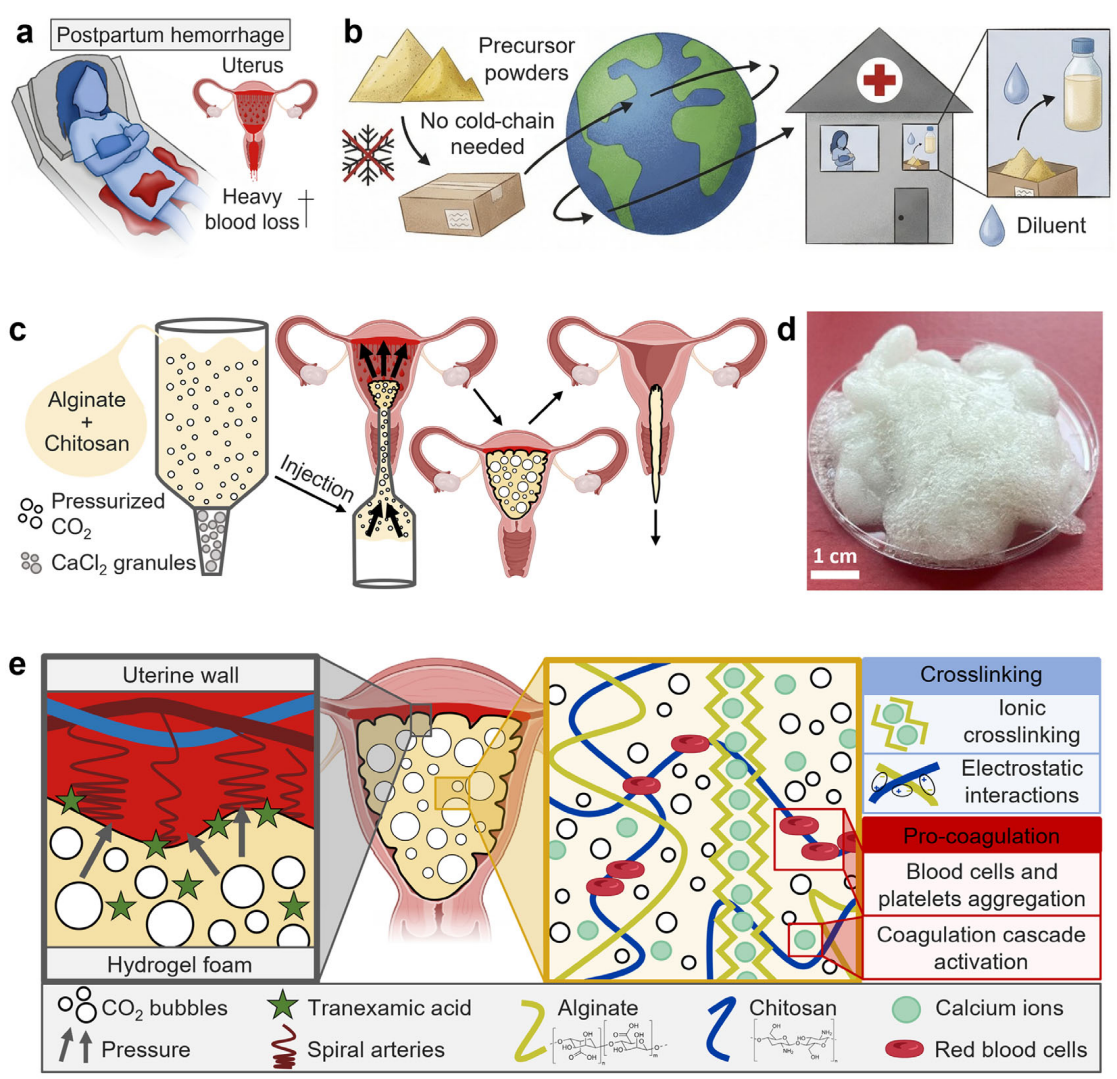
Hydrogel foam composition, generation, delivery and mechanism of action. (a) Scheme of postpartum hemorrhage, defined as an excessive blood loss after childbirth that can lead to death. (b) Scheme of compatibility of the precursor materials for transport without a cold‐chain and easy reconstitution in low‐resource settings. (c) Scheme of the application and removal of the hydrogel foam inside of the uterine cavity. Created with BioRender.com. (d) Macroscopic image of the hydrogel foam after complete expansion and crosslinking. (e) On the left side, scheme of the mechanisms of action to stop postpartum hemorrhage through pressure exertion on the spiral arteries and tranexamic acid release. On the right side, scheme of hydrogel foam composition (yellow frame), crosslinking mechanisms based on ionic crosslinking and electrostatic interactions (blue frame) and interactions with blood components (red frame). Created with BioRender.com.

To address the clinical challenges of PPH (Figure 1a), we developed an injectable hemostatic hydrogel foam that can be formed in situ and deployed even in low‐resource settings. The foam's network is composed of chitosan and alginate, formulated in dry form and only reconstituted with water and acetic acid upon application (Figure 1b). This up‐on‐use reconstitution enables transport of the precursors without the need for ensuring a cold‐chain, making the concept compatible with use in low‐resource settings. An applicator tube connected to a cream‐whipper‐inspired pressurizable device containing the precursors is inserted through the vaginal canal and the cervix into the uterine cavity (Figure 1c). During extrusion of the precursors, calcium ions from dissolution of calcium chloride (CaCl_2_) granules present in the applicator tube induce ionic crosslinking of alginate, and electrostatic interactions between chitosan and alginate reinforce the structure. Carbon dioxide (CO_2_) dissolved in the precursor solution drives self‐expansion of the hydrogel foam (Figure 1d) upon extrusion, creating gas bubbles that rapidly fill the uterine cavity and conform to its irregular geometry. Expansion of CO_2_ bubbles applies pressure on the uterine walls and compresses the spiral arteries, while hemostatic agents such as tranexamic acid can be incorporated for localized drug delivery (Figure 1e). The positively charged primary amines on chitosan polymer chains are known to attract and aggregate blood components to support clot formation [26]. Calcium ions play an essential role in coagulation cascade, and have been used in other hemostatic materials to activate coagulation [27]. Free calcium ions in the hydrogel foam can interact with blood and promote clot formation. Thus, the hydrogel foam combines mechanical tamponade with chemical coagulation support.

### 2.2 Hydrogel Foam Formulation and Characterization

**FIGURE 2:**
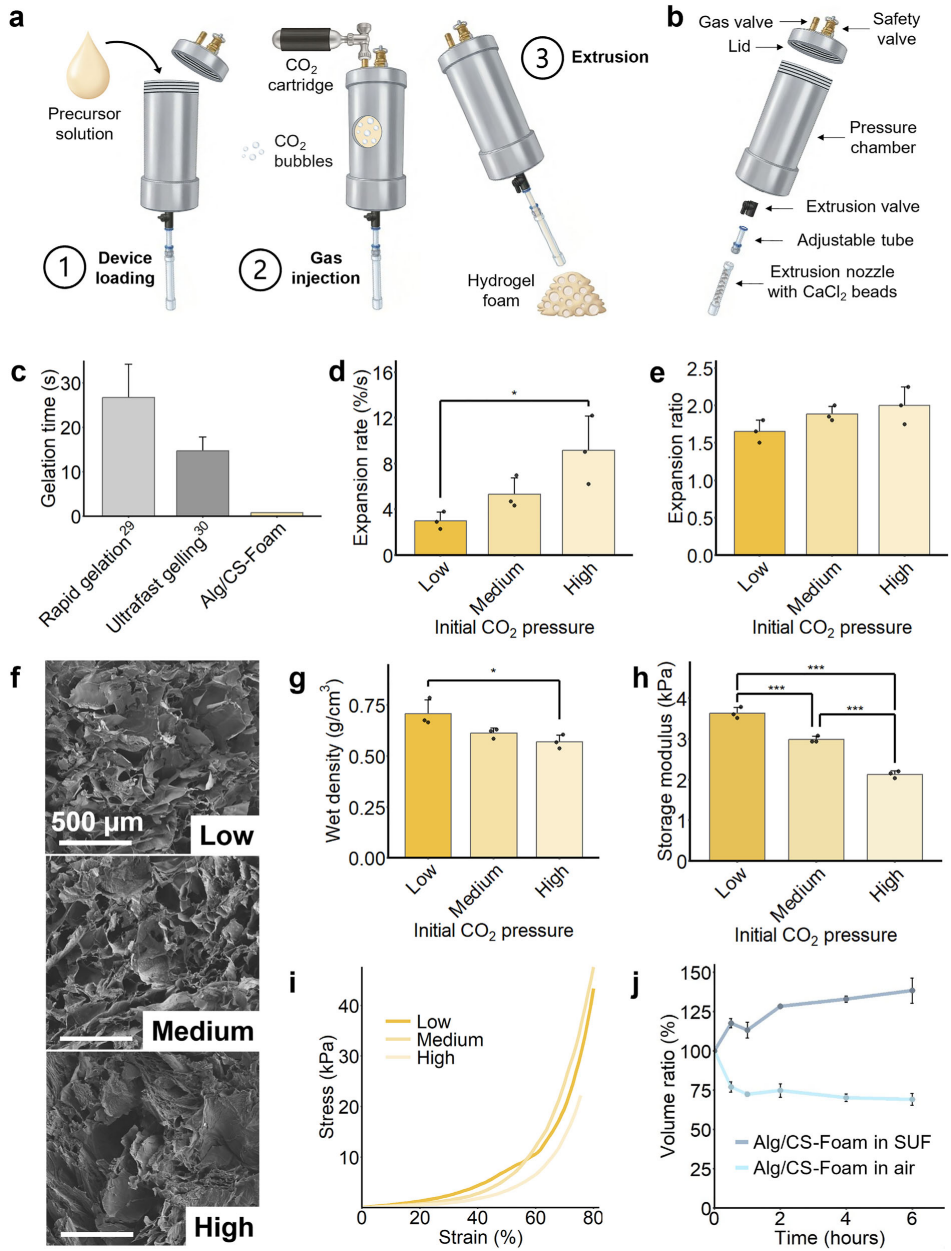
Hydrogel foam fabrication and characterization. (a) Scheme of hydrogel foam fabrication using the custom‐made fabrication device filled with precursor solution followed by CO_2_ gas injection, and extrusion of the hydrogel foam. (b) Scheme of custom‐made foam fabrication device. (c) Gelation time of the hydrogel foam in comparison with gelation times of other materials from the literature described with “rapid” [29] and “ultrafast” [30] gelation. (d) Expansion rate of the hydrogel foam showing tunability of the gas expansion kinetics during extrusion with Low, Medium, and High initial pressures (*n* = 3). (e) Expansion ratio of the hydrogel foam showing tunability of the gas expansion after extrusion with Low, Medium, and High initial pressures (*n* = 3). (f) Cross‐sectional SEM images of hydrogel foams with Low, Medium, and High initial pressures. (g) Wet foam density of the hydrogel foam after extrusion with Low, Medium, and High initial pressures (*n* = 3). (h) Storage moduli of the hydrogel foams with Low, Medium, and High initial pressures showing tunability of mechanical properties (*n* = 3). (i) Representative compressive stress‐strain curve of the hydrogel foam after extrusion with Low, Medium, and High initial pressures. (j) Volume ratio of the hydrogel foam fabricated with Medium initial pressure and left in air or soaked in simulated uterine fluid (SUF) over 6 h at 37°C (*n* = 3). Data are presented as mean ± standard deviation. Significance and *p*‐values were determined using ANOVA test followed by Tukey's HSD test. NS *p*>0.05, * *p* < 0.05, ** *p* < 0.01, *** *p* < 0.001.

The formulation and application method were optimized to enhance foam properties and delivery, guided by foam property assessments. As mentioned, the hydrogel foam can be prepared using a fabrication approach designed for field compatibility based on the mixing of an alginate/chitosan (Alg/CS) precursor with a calcium ion source, followed by CO_2_ driven foaming (Figure 2a). Different variations of foams were prepared by pressurizing CO_2_ into the precursor solution using a custom‐made foaming device inspired by a cream whipper device (Figure 2b and Figures S1 and S2). We first added a CaSO_4_ slurry to the precursor solution as a source of calcium ions with a passive mixer placed inside of the nozzle (Figure S3). It was hypothesized that the shear caused by the mixer would accelerate CaSO_4_ dissolution and enable rapid crosslinking [28]. However, this method led to insufficiently stable hydrogel foams due to limited crosslinking in the timeframe desired and was therefore dismissed. To achieve better crosslinking, we extruded the Alg/CS precursor through a nozzle containing CaCl_2_ granules, sourcing Ca^2+^ from CaCl_2_ dissolution (Figure S3). Using this approach, after extrusion and complete crosslinking, the resulting Alg/CS‐Foam remained stable. The granules did not obstruct the device as the foam could be continuously extruded without any problem. The optimal concentration of 2% alginate and 1% CS were chosen by comparing the resulting volume of stable hydrogel foam and the volume of remaining liquid precursor solution after extrusion of precursor solutions with various concentrations of alginate and CS (Figure S4). The final concentration of calcium ions in the hydrogel foam was measured for different initial precursor volumes (Figure S5). For comparison, hydrogel foam formulation containing alginate only (Alg‐Foam) was used to investigate the effect of chitosan addition on diverse hydrogel foam properties. Rheological measurements showed that Alg/CS‐Foam formed a stable structure, while Alg‐Foam exhibited liquid‐like behavior over time (Figure S6). The degradation profile of Alg‐Foam and Alg/CS‐Foam over 28 days showed a reduced weight loss in Alg/CS‐Foam, suggesting an increase in network stability when incorporating electrostatic crosslinking through alginate‐chitosan interactions (Figure S7). These results confirm that the network is reinforced by the addition of chitosan polymer chains. Importantly, in situ‐generated hydrogel‐based hemostatic materials should exhibit rapid gelation behaviors to stop the bleeding as fast as possible and to avoid being displaced by the blood flow. Compared to other materials with gelation characterized as “rapid” [29] and “ultrafast” [30], the proposed hydrogel foam presents significantly faster crosslinking, with a gelation time of around 1 s (Figure 2c and Videos S1–S3). The reconstitution workflow is depicted in Figure S8 and was tested by multiple trained and untrained operators to assess the robustness of the procedure and to measure the preparation time. The scheme shows the time needed for each step. Batch reproducibility was tested with two different lots of each polymer and is summarized in Table S1. The success rate over 10 preparations was 8/10 due to one technical failure of the device, and one improper foaming of the precursor. Storage stability of the dry precursor was tested by fabricating the hydrogel foam from powders stored for 1.5 years in Eppendorf tubes at ambient conditions and is reported in Table S1 as well.

Mechanical and physical tunability of hydrogel‐based materials are widely used to optimize the material properties for a particular application. In the context of PPH, the hydrogel foam fabrication was optimized based on the advice of an experienced gynecologist to obtain desired gas expansion and stability. Three different initial pre‐extrusion equilibrium pressures inside the fabrication device, hereafter referred to as initial pressures, were used to assess the tunability of the physical and mechanical properties of the material. Initial pressures of 4, 5, and 6 bar were tested, and are hereafter referred to as “Low”, “Medium”, and “High”, respectively. The expansion kinetics were determined by measuring the expansion rate of the foam, defined as the percentage of final volume per second (Figure 2d). A higher initial pressure led to faster expansion rate, ranging from 2.98 ± 0.75 to 9.13 ± 3.00%/s from Low to High initial pressure, respectively. The ratio between final hydrogel foam volume and initial precursor solution volume was used to measure the expansion ratio (Figure 2e). Increasing the initial pressure from Low to High led to an increase in the expansion ratio from 1.65 ± 0.15 to 2.00 ± 0.25. Therefore, the volume ratio of gas to hydrogel phase was higher with higher initial pressure. Despite no significant difference, the trend shows that varying the initial pressure allows for the control of gas expansion after extrusion.

In addition, the hydrogel foams were imaged using SEM to resolve the microscale structure at different initial pressures, with the limitation of artifacts introduced by drying (Figure 2f). The structures were highly porous, with larger pores observed in the samples fabricated with High initial pressure, suggesting the presence of larger gas bubbles. This was confirmed by wet density measurements of the hydrogel foam, which decreased from 0.71 ± 0.07 to 0.57 ± 0.03 g/cm^3^ when fabricating the foam with Low and High initial pressure, respectively (Figure 2g). The tunability of mechanical properties was assessed through rheological and compressive stress measurements (Figure 2h,i). Hydrogel foam with Low, Medium, and High initial pressures showed storage moduli (G’) of 3.63 ± 0.14, 2.98 ± 0.08, and 2.12 ± 0.09 kPa, respectively. G’ were inversely proportional to the initial pressures, leading to softer hydrogel foam when increasing initial pressure. The compressive stress of the hydrogel foams was measured and compared over different initial fabrication pressures (Figure 2i). At lower strain (<55%), the stress response was consistent with the rheological measurements, with Low, Medium, and High initial pressures showing progressively decreasing compressive stress of 2.25 ± 0.52, 2.10 ± 0.80 and 1.21 ± 0.23 kPa at 30% strain, respectively. At higher strain, the foam fabricated with Medium initial pressure exhibited the most pronounced strain‐stiffening, reaching the highest stress under large compression. The hydrogel foam fabricated with High initial pressure tended to tear apart due to weaker mechanical properties. Low and Medium initial pressures resulted in equally stable hydrogel foam. Alg/CS‐Foam fabricated with Medium initial pressure was selected over foam produced with Low initial pressure due to its higher expansion ratio and better mechanical properties. The hydrogel foam dimensional stability was assessed in air and in simulated uterine fluid (SUF) (Figure 2j). Alg/CS‐Foam swelled in SUF, reaching 138.34 ± 7.96% of its initial volume after 6 h. The volume of Alg/CS‐Foam decreased to 69.15 ± 3.72% of the initial volume after 6 h in air. The decrease in volume when exposed to air suggest that the hydrogel foam slowly collapses over time. However, the hydrogel foam will be in constant contact with the wet uterine environment, and therefore a volume increase can be expected throughout the duration of the tamponade.

### 2.3 In Vitro Hemostatic Activity and Hemocompatibility

**FIGURE 3:**
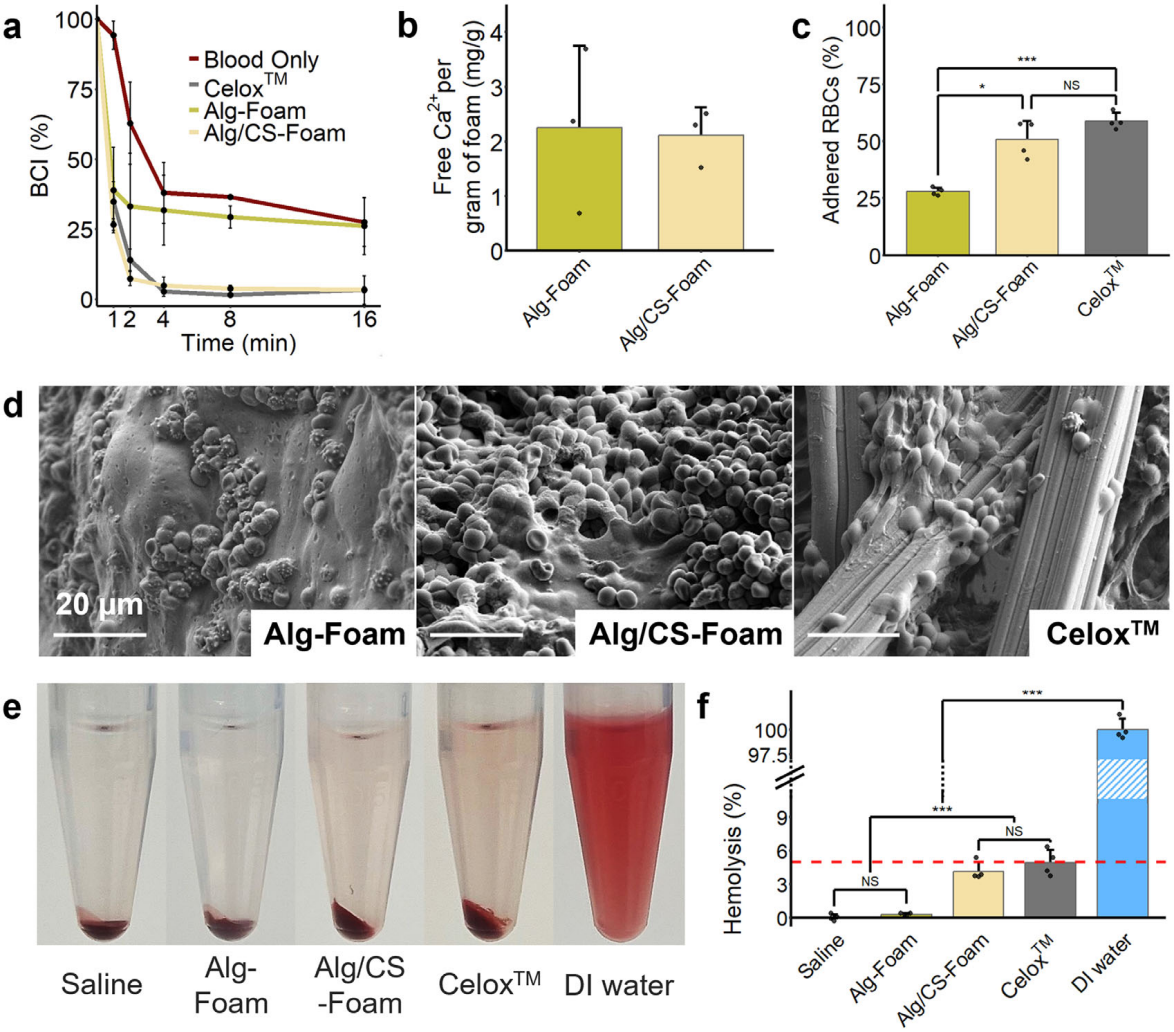
In vitro hemostatic activity and hemocompatibility of hydrogel foams and Celox. (a) Blood coagulation index of Control (Blood Only), Celox, Alg‐Foam, and Alg/CS‐Foam (*n* = 3). (b) Mass of free Ca^2+^ per gram of Alg‐Foam and Alg/CS‐Foam after 6 h in DI water (*n* = 3). (c) Percentage of adhered RBCs on Alg‐Foam, Alg/CS‐Foam, and Celox (*n* = 4). (d) SEM images of blood cells (RBCs and platelets) adhesion on Alg‐Foam, Alg/CS‐Foam, and Celox. (e) Pictures from hemocompatibility assay of saline, Alg‐Foam, Alg/CS‐Foam, Celox, and DI water. (f) Hemolysis ratio of Saline, Alg‐Foam, Alg/CS‐Foam, Celox, and DI water (*n* = 4). Dotted red line represent the hemocompatibility threshold. Data are presented as mean ± standard deviation. Significance and p‐values were determined using ANOVA test followed by Tukey's HSD test. NS *p*>0.05, * *p*<0.05, ** *p*<0.01, *** *p*<0.001.

Pro‐coagulant activity and blood biocompatibility are requirements for any hemostatic materials. To assess the hemostatic activity of the hydrogel foam and to investigate the role of calcium ions and chitosan, Alg‐Foam and Alg/CS‐Foam were compared to commercially available chitosan gauze (Celox PPH). The blood coagulation index (BCI), defined as the capacity of a material to enhance blood clot formation, was compared between the hydrogel foams, Celox, and a control without material (Blood only; Figure 3a). After 1 min of contact with blood, the BCIs of Alg‐Foam, Alg/CS‐Foam, and Celox were lower than the control, showing the capacity of the materials to accelerate coagulation. After 16 min, a lower BCI was observed for Alg/CS‐Foam and Celox gauze, compared to Alg‐Foam and the control. These results suggest that the free calcium ions accelerate coagulation, and the addition of chitosan in the hydrogel foam reinforces the blood clot on the Alg/CS‐Foam, reaching efficacies similar to Celox gauze. Calcium ions act as co‐factors for the activation of factors IX and X, as well as for the activation of prothrombin into thrombin, that will then activate fibrinogen to form a fibrin network [31]. Therefore, the presence of free calcium ions in Alg‐Foam and Alg/CS‐Foam activate the coagulation cascade and accelerate blood clot formation. Free Ca^2+^ release was measured after soaking Alg/CS‐Foam in DI water for 6 h (Figure 3b). Per gram of hydrogel foam, 2.2 ± 1.5 mg, and 2.1 ± 0.5 mg of free Ca^2+^ was released from Alg‐Foam and Alg/CS‐Foam, respectively. Chitosan acts independently from the plasma coagulation cascade, by adhering, activating, and aggregating blood cells and platelets [32]. The addition of chitosan in Alg/CS‐Foam compared to Alg‐Foam appears to reinforce the blood clot. The interactions between red blood cells (RBCs) and the materials were then quantified by contacting concentrated RBCs with the surface of the materials and measuring the proportion of adhered RBCs. The percentage of adhered RBCs was significantly higher in Alg/CS‐Foam (50.79 ± 7.99%) and Celox gauze (58.80 ± 3.66%) compared to Alg‐Foam (27.88 ± 1.68%) (Figure 3c). SEM images show the morphology of the blood cells adhered onto the surface of the hydrogel foams and the gauze (Figure 3d). A large fraction of the RBCs are aggregated onto all the materials and have a rounder shape typically observed in blood clots, compared to their native biconcave shape [33]. These results show that the hydrogel foams promoted RBCs aggregation and blood clotting with Alg/CS‐Foam hydrogel foam having a comparable efficacy to chitosan‐based commercially available hemostatic gauze. In addition to coagulation, hemocompatibility, and especially the absence of hemolysis, is essential in the development of hemostatic materials. The osmolality of the hydrogel foams was determined by immersing Alg‐Foam and Alg/CS‐Foam in DI for 6 h and measuring the osmolality of the resulting solution (Figure S9). The osmolality of Alg‐Foam and Alg/CS‐Foam were 242.21 ± 93.19 and 238.58 ± 29.05 mOsm kg^−1^, respectively. These values correspond to hypotonicity compared to the physiological range of plasma tonicity (275–295 mOsm kg^−1^), which could compromise the RBCs integrity and result in hemolysis [34]. To test whether the hypotonicity of the hydrogel foams affects RBCs integrity, the hemolysis ratio of hydrogel foams was calculated based on the incubation of blood with the materials and subsequent measurement of the absorbance of the solution to determine the amount of released hemoglobin. The hemolysis ratio of commercially available hemostatic gauze was determined as a control. Images of the supernatant after incubation showed no significant coloring compared to complete hemolysis in DI water (Figure 3e). Quantitative measurements of hemolysis ratio are presented in Figure 3f. Alg‐Foam showed the least hemolysis, with a ratio of 0.53 ± 0.21%. Alg/CS‐Foam hemolysis ratio was higher (4.87 ± 1.30%), and close to the standard 5% threshold used in the literature but was lower than commercially available Celox (5.89 ± 1.76%). These data suggest no significant hemolysis for the hydrogels compared to commercially available hemostatic gauze.

### 2.4 Hydrogel Foam Biocompatibility

**FIGURE 4:**
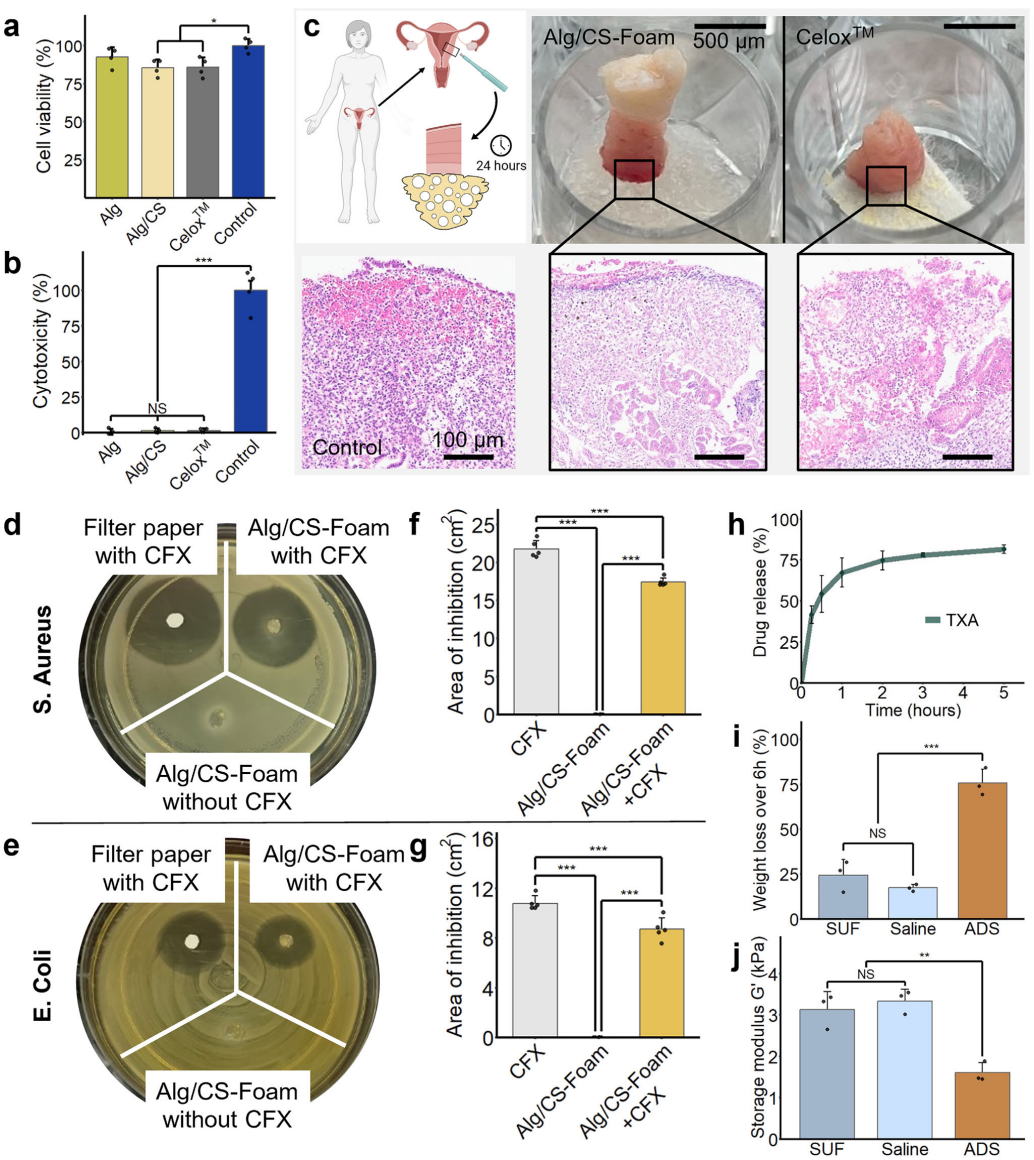
Biocompatibility, antimcriobial and hemostatic drug release, and in vitro accelerated degradation. (a) Cell viability assay and (b) cytotoxicity assay of Alg‐Foam, Alg/CS‐Foam and Celox on human endometriotic cells showed good biocompatibility (*n* = 4). (c) Scheme of experiment setup of tissue‐material interactions on ex vivo human uterine endometrium biopsies, macroscopic images of experimental setup for tissue‐material interaction with the hydrogel foam (Alg/CS‐Foam) and Celox, and histological micrographs of tissue after 24 h contact with no material (Control), Alg/CS‐Foam, and Celox. Created with BioRender.com. (d,e) Macroscopic images of growth inhibition assay with filter paper loaded with cefuroxime (CFX), Alg/CS‐Foam, and CFX‐loaded Alg/CS‐Foam on (d) *S. aureus* and (e) *E. coli*. f‐g Quantitative analysis of area of (f) *S. aureus* and (g) *E. coli* growth inhibition with CFX‐loaded filter paper (CFX), Alg/CS‐Foam and CFX‐loaded Alg/CS‐Foam (Alg/CS‐Foam+CFX) (*n* = 5). (h) Drug release curve of Alg/CS‐Foam loaded with tranexamic acid (TXA) (*n* = 3). (i) In vitro degradation of Alg/CS‐Foam in simulated uterine fluid (SUF), 0.9% NaCl (Saline), and accelerated degradation solution (ADS) after 6 h (*n* = 3). (j) Storage modulus of Alg/CS‐Foam after 6 h incubation in SUF, Saline, and ADS (*n* = 3). Data are presented as mean ± standard deviation. Significance and *p* values were determined using ANOVA test followed by Tukey's HSD test. NS *p*>0.05, * *p*<0.05, ** *p*<0.01, *** *p*<0.001.

Hydrogel foams were assessed for cytocompatibility and compared to Celox hemostatic gauze. Cell culture media was incubated for 24 h with hydrogel foams or gauze, and human endometriotic cells were incubated with the resulting culture media for another 24 h. Then, viability of human endometriotic cells was determined through a metabolic activity assay (CellTiterGlo), and a lactate dehydrogenase (LDH) assay was performed to assess the potential cytotoxicity of the materials. No significant decrease in cell viability and no significant cytotoxicity was observed in any of the conditions (Figure 4a,b). Alg/CS‐Foam and Celox were put in contact with biopsies from human uterine tissue over 24 h to investigate potential adhesion of the materials to the endometrium (Figure 4c). For the hydrogel foam and for Celox, no obvious sign of tissue adhesion was observed as no residual material appeared on the histological scans. For further confirmation of the compatibility, an in vivo study was performed (vide infra).

### 2.5 Hydrogel Foam Antimicrobial and Hemostatic Drug Release

To assess the feasibility of drug release and topical treatment, the hydrogel precursor solution was mixed with different drugs, Cefuroxime (CFX) on the one hand, and tranexamic acid (TXA) on the other hand. To assess the addition of antimicrobial agents, CFX, a broad‐spectrum cephalosporin antibiotic routinely used to prevent peripartum infections, was included into the foam [35]. Incorporation of CFX in the proposed hydrogel foam may allow for topical release of the antibiotic to prevent endometritis. An in vitro bacterial growth assay was performed on Gram‐positive (*S. aureus*) and Gram‐negative (*E. coli*) bacteria to determine the capacity of Alg/CS‐Foam to release CFX (Figure 4d–g). The area of inhibition was compared between Alg/CS‐Foam and CFX‐loaded Alg/CS‐Foam (Alg/CS‐Foam+CFX). Filter paper soaked with a CFX solution was used as a control. The results show that Alg/CS‐Foam alone did not prevent the growth of *S. aureus* nor *E. coli*, while the area of *S. aureus* growth inhibition of Alg/CS‐Foam+CFX (17.41 ± 0.57 cm^2^) was comparable to the area of inhibition of the control (21.74 ± 1.14 cm^2^). A similar trend was observed with *E. coli* growth inhibition, where no inhibition was observed for Alg/CS‐Foam alone, and a comparable area of inhibition was measured when using Alg/CS‐Foam+CFX (8.72 ± 0.91 cm^2^) and the CFX control (10.81 ± 0.59 cm^2^). Therefore, it can be concluded that CFX was successfully incorporated and released from Alg/CS‐Foam and the antibacterial activity of CFX against Gram‐positive and Gram‐negative bacteria was preserved. Hemostatic agents such as TXA are routinely administered systemically for the treatment of postpartum hemorrhage and have also been reported for topical application [36, 37] The drug release capacity of Alg/CS‐Foam was investigated for TXA through fluorescent labelling. Alg/CS‐Foam showed a sustained release of TXA, reaching 81.65 ± 2.55% after 5 h (Figure 4h). These results show the hemostatic drug release capacity of Alg/CS‐Foam for the delivery of topical hemostatic agents.

### 2.6 Hydrogel Foam Degradation and Excretion

In the management of PPH, degradable materials are advantageous to avoid a second surgical intervention and prevent bacterial colonization of the biomaterial. In the case of PPH, the material needs to degrade in a timely manner to allow the uterus to contract back to its native size and shape. Recent studies suggest that intrauterine tamponade should be removed after 6–12 h [38, 39, 40]. Mothers experiencing PPH are usually staying at the hospital for 2–3 days [41]. Therefore, the ideal tamponade should be stable for at least 6 h and be degradable within 1.5 days to avoid prolongation of the hospital stay. Enzymatic degradation of alginate‐based materials by alginate lyase is a common method to accelerate degradation [42]. An accelerated degradation solution (ADS) based on alginate lyase was tested to accelerate the degradation of Alg/CS‐Foam and the weight loss was compared to degradation in normal saline (Saline) and simulated uterine fluid (SUF). Figure 4i shows the weight loss of Alg/CS‐Foam incubated for 6 h in the different solutions. Alg/CS‐Foam showed significantly more degradation when incubated in ADS than in Saline, or SUF. A weight loss of 75 ± 8% was observed after 6 h incubation in ADS, offering a suitable mechanism for the timely removal of the hydrogel foam. For painless removal through the cervix and vaginal canal, the hydrogel foam needs to be soft. The storage modulus of Alg/CS‐Foam after 6 h incubation in ADS was measured and compared to the storage modulus of samples incubated for 6 h in SUF or saline. Figure 4j shows the storage moduli of samples after 6 h incubation in SUF, saline, and ADS. The storage moduli were significantly lower after incubation in ADS (1.60 ± 0.25 kPa) compared to incubation in SUF (3.13 ± 0.43 kPa) and saline (3.34 ± 0.29 kPa). These results suggest that the ADS can effectively degrade and soften the hydrogel foam to allow for facilitated and timely excretion through the vaginal canal. The accelerated degradation solution and degradation products were assessed for cytocompatibility on normal human dermal fibroblasts (NHDFs) (Figure S10). Alg/CS‐Foam was soaked for 6 h in ADS and cells were cultured with the resulting solution. Cells were also cultured in ADS and in 0.9% NaCl as controls. The cell viability showed no decrease and there was no sign of cytotoxicity. The pH of the foam was determined by immersing Alg‐Foam and Alg/CS‐Foam in DI water over 6 h and measuring the pH of the resulting solution at different time points (Figure S11). The pH ranged from 5.2 to 6.2, a mildly acidic range comparable to the saline (∼pH 5.5) routinely used for saline infusion sonohysterography, suggesting good tolerability within the uterine cavity [43]. The residual acetic acid was determined by immersing Alg/CS‐Foam in DMSO containing 0.3 wt.% maleic acid (MA) and in D_2_O containing 0.5 wt.% potassium hydrogen phthalate (KHP) and calculating the released acetic acid based on the NMR spectra (Figure S12). The content of residual acetic acid per gram of Alg/CS‐Foam was 4.76 ± 0.13 mg/gr. Comparable levels have been reported for other hydrogels intended for intrauterine application and tested in vivo [44, 45] and 5% acetic acid solutions are commonly used for established gynecological procedures such as visual inspection of the cervix for cervical cancer diagnosis [46].

### 2.7 In Vitro Postpartum Hemorrhage Model

**FIGURE 5:**
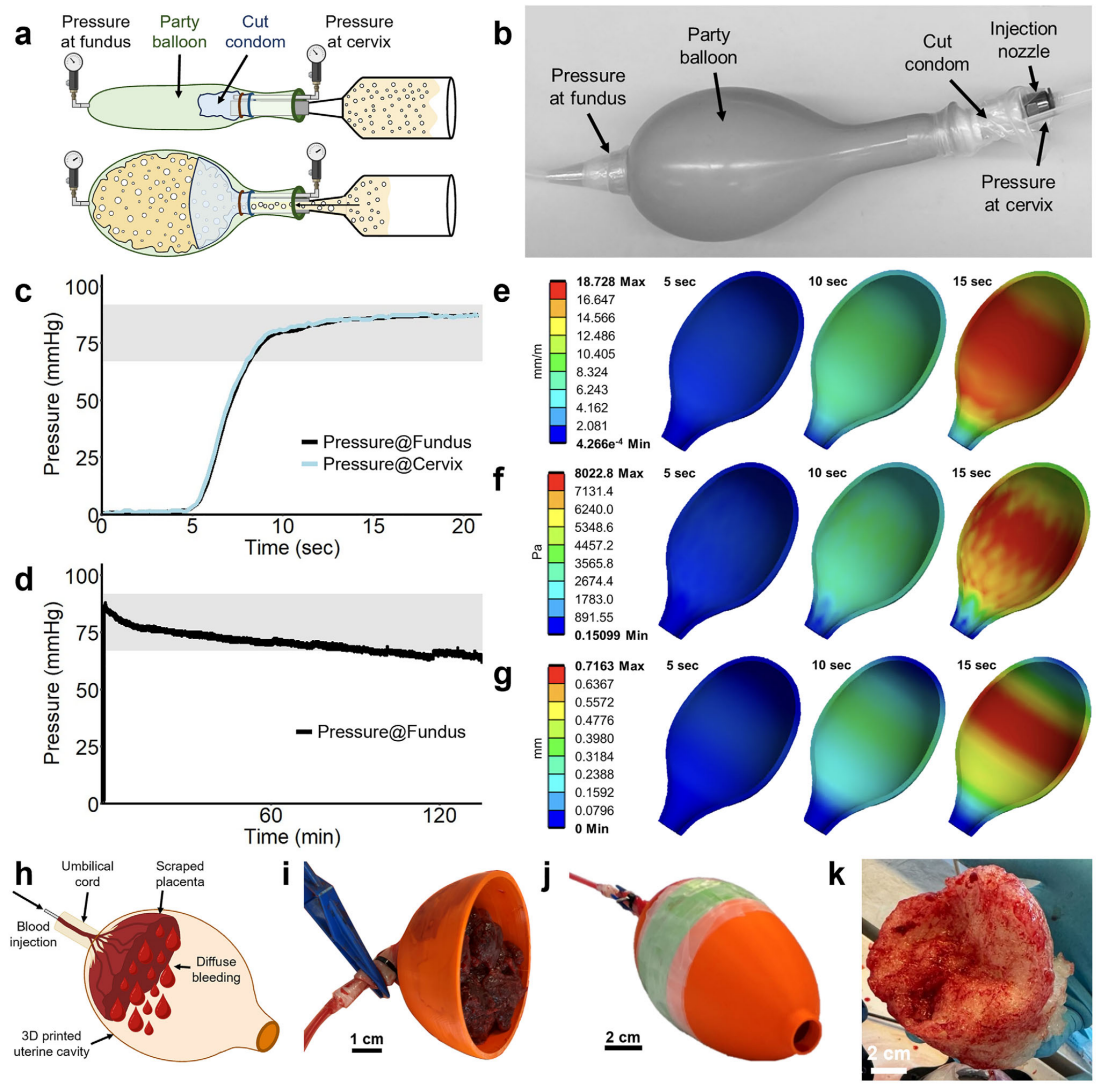
In vitro postpartum hemorrhage model and hydrogel foam expansion simulation. (a) Scheme of balloon‐based in vitro PPH model. (b) Picture of PPH model with pressure sensing setup. (c) Intrauterine pressures measured at the cervix and at the fundus correlate during hydrogel foam injection. (d) Sustained fundal pressure for over 120 min. (e–g) Two‐way fluid–structure interaction (FSI) simulation in ANSYS of strain (e) and stress (f) on the uterine wall, and deformation of the uterine wall (g) during hydrogel foam extrusion. (h) Scheme of bleeding model with placenta. (i) Picture of fundal part of bleeding model. (j) Picture of closed bleeding model. (k) Hydrogel foam after injection in the bleeding model.

Exerting sufficient pressure on the uterine wall is essential to compress the spiral arteries and control postpartum bleeding. Minimally invasive pressure monitoring, particularly during injection, is essential to ensure that sufficient pressure is applied, and to avoid overpressure and potential damage to the tissues. The intraluminal pressure of the Bakri balloon usually reaches 67–92 mmHg at the end of balloon inflation [10]. This range was used as a reference for the targeted pressure range to reach during hydrogel foam injection. To test the pressure exertion capacity of the hydrogel foam and the feasibility of minimally invasive pressure monitoring from the cervix, an in vitro model was established using two layers of party balloons to mimic the uterine cavity (Figure 5a,b). The model includes a pressure sensor at the tip of the balloon, approximating the location of the fundus, and therefore the bleeding site, to assess the compression of spiral arteries. A second pressure sensor was placed at the neck of the balloon to investigate minimally invasive pressure monitoring from the cervix. An open condom was placed around the extrusion nozzle and expanded upon injection of the hydrogel foam to seal the cervix and allow for intrauterine pressure to build up. This cervix sealing mechanism was discussed with an experienced gynecologist and is intended for application also in the human body. To assess if the pressure at the fundus can be accurately measured from the pressure sensor placed at the cervix, readings of pressure sensors at the fundus and at the cervix were recorded simultaneously and compared. Figure 5c presents the pressure readings at the fundus and at the cervix. The perfect overlap of the two pressures confirms the feasibility of a pressure reading at the cervix that will accurately represent the pressure exerted at the fundus, and thus at the bleeding site. In addition, it supports the possibility for minimally invasive monitoring of the pressure from the cervix to ensure safety during hydrogel foam injection. Intrauterine tamponades are usually kept in place for maximum 24 h, but recent studies suggest that 6–12 h are already sufficient [38, 39, 40] The hydrogel foam showed a capacity to sustain sufficient intrauterine pressure for more than 2 h (Figure 5d). To sustain the pressure for a longer period, more hydrogel foam can be injected. These repeated injections showed restoration of the targeted pressure and allowed for intrauterine pressure tuning (Figure S13). The hydrogel foam was then injected in the in vitro uterine model immersed in SUF (Figure S14). The intrauterine pressure for appropriate tamponade was reached and could be sustained for an hour, confirming that the hydrogel foam can be injected in the simulated uterine environment. In contrast to the foam, the Bakri balloon cannot conform to the uterine cavity shape, which can greatly reduce its efficacity. The proposed hydrogel foam with in situ formation is designed to conform to the irregular contours of the uterus, facilitating optimal material‐tissue interactions, effective pressure application, and hemostasis. A two‐way fluid–structure interaction (FSI) simulation of the hydrogel foam expansion inside of the uterus was conducted in ANSYS. The pressure exerted on the uterine wall by CO_2_ expansion over time was simulated to determine the dynamics of the uterine wall in terms of strain, stress, and deformation (Figure 5e–g). The expansion of CO_2_ allows Alg/CS‐Foam to fill the cavity entirely and apply pressure on the wall. In the simulation, the uterus was represented as an ellipsoid, and the uterine wall was modelled as a smooth surface. To obtain a model closer to reality, a physical PPH model mimicking the irregularities of the uterine wall as well as the diffuse bleeding was developed using human placenta (Figure 5h–j).

The maternal side of the placenta, which attaches to the uterine wall, closely resembles the structure of the bleeding surface in PPH and was used to mimic the irregular uterine wall surface. To replicate heavy diffuse bleeding, the placenta was scraped, and blood was perfused to the placenta through the umbilical artery. Despite the perfused blood, the hydrogel foam formed a stable structure that conformed to the irregular shape of the scraped placenta (Figure 5k). These results suggest that despite the presence of heavy bleeding, the system allows for in situ formation of the hydrogel foam and ensures the conformation of the hydrogel foam to the irregular contours of the uterine cavity. The on‐demand degradation was tested in the in vitro uterus model and the weight loss was recorded (Figure S15). After a single injection of the ADS and 6 h incubation at 37°C, the hydrogel foam lost 41.43 ± 21.18% of wet weight, compared to 20.80 ± 10.13% when exposed to SUF only. The hydrogel foam was partially degraded by the ADS, and further optimization and device development are needed to ensure complete removal of the material from the uterine cavity.

### 2.8 Ex Vivo Swine Uterus Model

**FIGURE 6:**
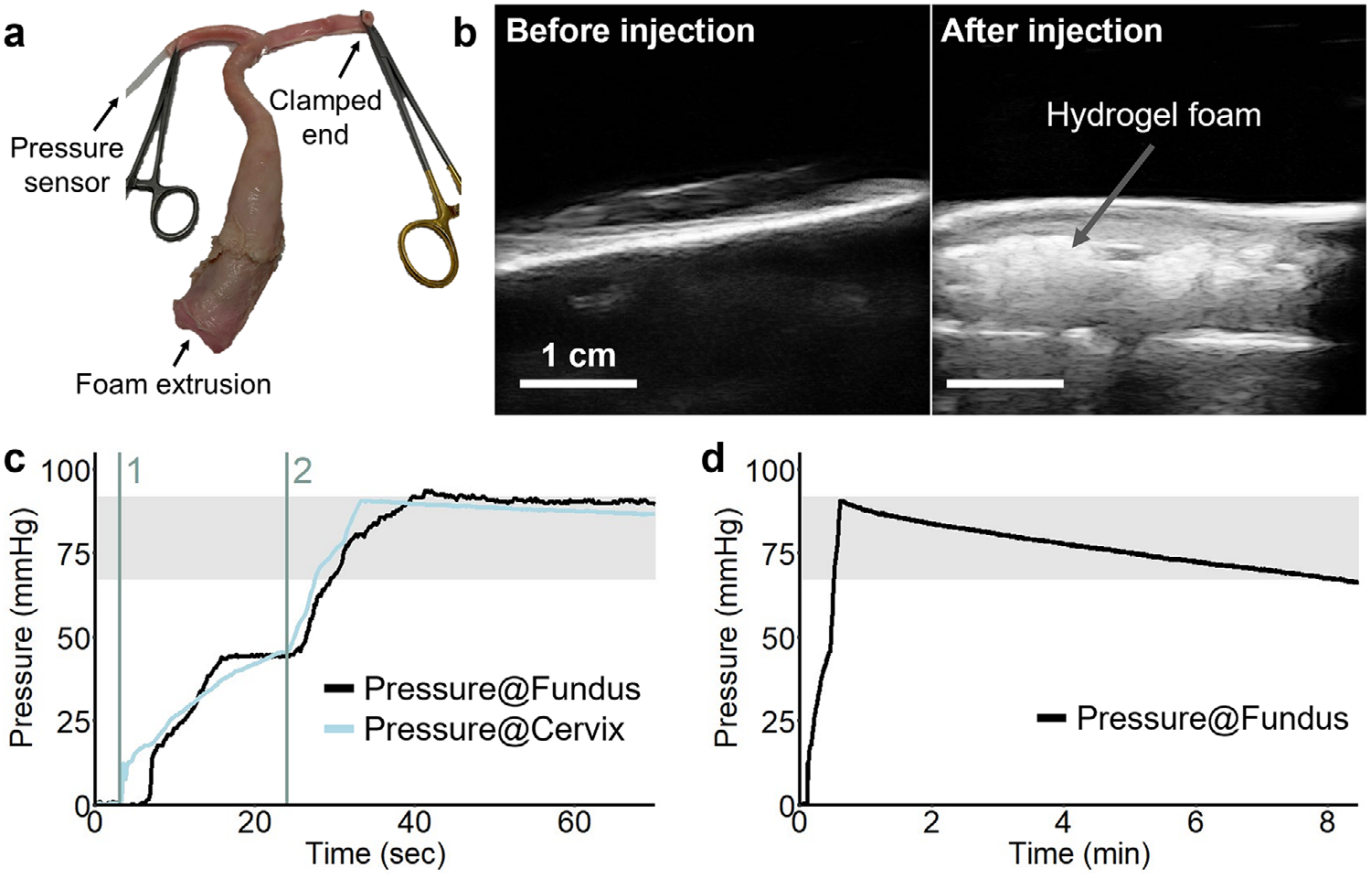
Hydrogel foam application in ex vivo swine uterus. (a) Annotated macroscopic image of ex vivo swine uterus model setup. Fundal pressure was measured in one of the horns while the other horn was clamped. (b) Ultrasound images before and after Alg/CS‐Foam injection. (c) Fundal pressure measurement during controlled step extrusion of Alg/CS‐Foam in ex vivo swine uterus. 1 and 2 represent the start of each extrusion step. Area in grey represents target pressure range of 67–92 mmHg. (d) Sustained fundal pressure measured after completion of the Alg/CS‐Foam extrusion in the swine uterus.

After validating the proposed material on the custom‐made in vitro models, the hydrogel foam was tested ex vivo using swine uterus to approximate human uterus anatomy and tissue mechanical properties. Ex vivo swine uteri were used to examine ultrasound imaging as a non‐invasive method to guide hydrogel foam injection, and to test the monitoring of sustained intrauterine pressure under biological tissue conditions (Figure 6a). The nozzle was inserted in the cervix through the vaginal canal and the hydrogel foam was injected as described before. Ultrasound images from the left horn were taken right above the cervix before and after hydrogel foam injection (Figure 6b). The hydrogel foam produced a bright ultrasound signal inside of the uterus compared to the empty uterus. The presence of gas bubbles within the hydrogel foam creates significant acoustic heterogeneity, resulting in increased echogenicity that facilitates the localization of the material. This result suggests that ultrasound imaging offers a suitable non‐invasive method to guide hydrogel foam injection and ensure adequate filling of the uterine cavity. Then, the feasibility of minimally invasive intrauterine pressure monitoring was evaluated by recording the pressure from the cervix and from the left horn. The intrauterine pressure was measured from the cervix using the same nozzle as in the in vitro balloon model and from the uterine horn using a tube connected to a pressure sensor and secured inside the horn. The right horn was clamped. Figure 6c shows representative pressure measurements during the hydrogel foam injection phase and the reaching of the target pressure range within minutes. The data also shows that the pressure in the horn, corresponding to the fundal pressure, could be reliably measured from the cervix to allow for minimally invasive intrauterine pressure monitoring. Finally, sustained pressure in the horn was measured for about 8 min (Figure 6d). The ex vivo experiments on the swine uterus demonstrate that the hydrogel foam can be clearly identified using non‐invasive ultrasound imaging, and that the minimally invasive intrauterine pressure measurement allows for monitoring of intrauterine pressure during the injection.

### 2.9 In Vivo Application and Hemostatic Properties

**FIGURE 7:**
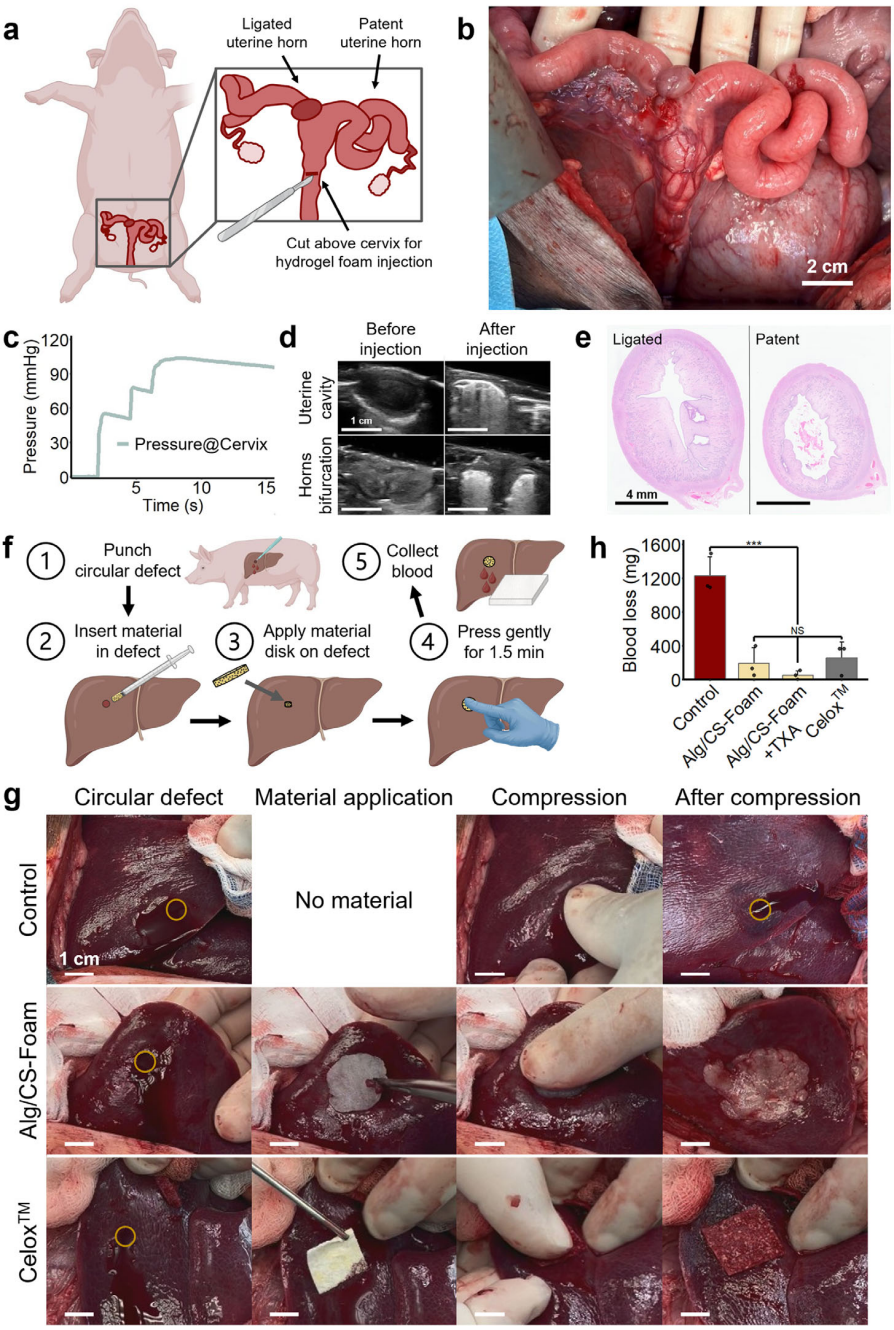
In vivo hydrogel foam application and hemostatic activity in swine model. (a) Scheme of in vivo study of hydrogel foam application in a swine uterus. Created with BioRender.com. (b) Macroscopic image of applied hydrogel foam in swine uterus. (c) Cervix pressure measurement illustrates step extrusion and time of application of hydrogel foam. (d) Ultrasound image of the swine uterine cavity and horns before and after injection of the hydrogel foam. (e) Histology of control horn vs horn with hydrogel foam. (f) Scheme of in vivo hemostatic activity study on circular liver defect. Created with BioRender.com. (g) Macroscopic images of the hemostatic effect of Control, Alg/CS‐Foam and Celox. Dotted circles outline the defect boundaries. (h) Blood loss measured after removal of the pressure for defect treated without material (Control), or with Alg/CS‐Foam, Alg/CS‐Foam and tranexamic acid (Alg/CS‐Foam+TXA), or Celox (*n* = 3). Data are presented as mean ± standard deviation. Significance and *p* values were determined using ANOVA test followed by Tukey's HSD test. NS *p*>0.05, * *p*<0.05, ** *p*<0.01, *** *p*<0.001.

To evaluate the proposed material under in vivo conditions, the application and hemostatic properties of the hydrogel foam were tested on a porcine model. First, the hydrogel foam was injected into the uterus of a swine to demonstrate anatomical feasibility through delivery, in‐situ expansion, and conformation within the relevant cavity geometry (Figure 7a). Ligation was used to prevent the hydrogel foam from filling one of the horns, thereby serving as a control. Figure 7b shows a macroscopic image of the uterus after complete hydrogel foam injection. Minimally invasive monitoring of the intrauterine pressure was carried out using the same system as for in vitro and ex vivo pressure monitoring. The hydrogel foam was injected in three steps, and the intrauterine pressure was measured from the cervix (Figure 7c). The injection was completed in less than 10 sec, and an intrauterine pressure of 104 mmHg was reached, which is comparable to successful Bakri balloon procedures [47]. B‐mode ultrasound was used for non‐invasive imaging of the foam during and after injection to confirm the proper filling of the uterine cavity. Figure 7d shows ultrasound images of the uterine cavity and the horns separation before and after injection. Thanks to the CO_2_ gas bubbles, the hydrogel foam can be clearly identified with ultrasound B‐mode imaging. The combination of minimally invasive intrauterine pressure monitoring and non‐invasive ultrasound‐based imaging allow for the control of safe, sufficient, and successful injection of the hydrogel foam into the uterine cavity. After the procedure, biopsies were taken from the control ligated horn, as well as the horn filled with hydrogel foam. Histological samples show no relevant damage to the epithelium of the endometrium after contact with the hydrogel foam (Figure 7e). The uterus is known to start involution soon after childbirth, where remodeling through necrosis and regeneration of the pregnant endometrium, or decidua, restores the uterus prepartum state [48]. These results suggest that the hydrogel foam does not cause damage to the uterine wall. A limited amount of reports describe surgically‐induced intrauterine bleeding, and there are no standard procedures to model postpartum hemorrhage in animals [49]. To confirm our findings in a hemorrhage scenario, a standard columniform defect on the liver was used to assess in vivo hemostatic properties of the hydrogel foam (Figure 7f). The defect was filled and covered with the material before applying gentle manual compression. After the compression was stopped, the blood loss was recorded using pre‐weighted surgical gauzes. The defects treated with Alg/CS‐Foam, Alg/CS‐Foam with tranexamic acid (Alg/CS‐Foam+TXA) and Celox all showed a significant reduction in blood loss compared to the control without material (Figure 7g,h and Figures S16 and S17). The blood loss was significantly reduced from 1229 ± 225 mg in the control group to 194 ± 185 mg when applying Alg/CS‐Foam, and to 52 ± 53 mg when adding tranexamic acid to Alg/CS‐Foam. Celox contained the bleeding to a blood loss of 260 ± 186 mg. This data suggests that the proposed hydrogel foam, with or without tranexamic acid, has similar hemostatic properties compared to the commercially available gauze. The in vivo experiments confirm the safe and successful intrauterine injection of the material, as well as the promising hemostatic properties of the hydrogel foam. However, the hydrogel foam was injected in a healthy, non‐postpartum swine without active bleeding, and the liver hemorrhage does not fully reproduce all aspects of postpartum hemorrhage blood loss. A key next step will be to further validate the technology in an atonic or actively bleeding postpartum uterine model.

## 3 Conclusions

In this work, we present an injectable hydrogel foam designed for field compatibility that forms and self‐expands in situ to rapidly control PPH, with a design specifically suited for low‐ and middle‐income country settings. The foam provides sustained, soft intrauterine pressure while offering dual hemostatic activity and localized drug delivery. Its mechanical properties enable effective tamponade without tissue damage, and in vitro studies with human blood confirmed robust coagulation and hemocompatibility. Accelerated, on‐demand degradation allows timely and facilitated removal after bleeding control. The material successfully delivered tranexamic acid and antibiotics, enabling local pharmacological support without reliance on systemic administration. Controlled pressure exertion and stability were demonstrated in vitro and ex vivo, supported by a minimally invasive pressure‐monitoring strategy that ensures safe deployment. The hydrogel foam reliably formed under high perfusion rates in a perfused placenta model of diffuse PPH and was successfully injected in vivo in a swine uterus with ultrasound‐guided visualization. No apparent tissue damage was observed by histology, and in vivo hemostatic efficacy was further confirmed in a liver bleeding model. Overall, this injectable hydrogel foam represents a new PPH management approach that integrates mechanical tamponade, coagulation support, pressure monitoring, and topical drug delivery in a single, easy‐to‐deploy system. By avoiding complex hardware, electricity, or advanced infrastructure (such as cold chain), the technology is inherently compatible with field use and low resource healthcare environments. Pending further evaluation, this approach has the potential to expand access to effective PPH care and meaningfully reduce global maternal health inequities.

## 4 Experimental

Alginic acid sodium salt (LMW, 12 000–80 000 Da, M/G ratio of 1.56), chitosan (LMW, 50 000–190 000 Da, ≥75% deacetylation), acetic acid, sodium hydroxide (NaOH), calcium chloride (CaCl_2_, granules, ≤7.0 mm), calcium sulphate (CaSO_4_), sodium chloride (NaCl), potassium chloride (KCl), calcium chloride (CaCl_2_, powder), sodium bicarbonate (NaHCO_3_), D‐glucose (glucose), sodium phosphate monobasic dihydrate (NaH_2_PO_4_), phosphate buffered saline (PBS), citrate‐phosphate‐dextrose solution (CPD), glutaraldehyde, ethanol (EtOH), alginate lyase, brain‐heart infusion (BHI), cefuroxime (CFX), tranexamic acid (TXA), fluorescamine, dimethyl sulfoxide (DMSO), deuterium oxide (D_2_O), maleic acid (MA) and potassium hydrogen phthalate (KHP) were purchased from Sigma‐Aldrich. Fresh porcine uteri were provided by a local slaughterhouse (SBZ Schlachtbetrieb Zürich AG) and were stored at −20°C in double sealed freezer bags. Normal human dermal fibroblasts (NHDFs) were purchased from Sigma‐Aldrich and endometrial cells (12Z) and materials used for cell culture were purchased from abm (Applied Biological Materials Inc., USA). Staphylococcus Aureus (ATCC 25923) and Escherichia Coli (ATCC 25922) were purchased from Leibniz Institute (DSMZ‐German Collection of Microorganisms and Cell Cultures GmbH, Braunschweig, Germany).

### 4.1 Hydrogel Foam Precursor Solution Preparation

To prepare the hydrogel foam precursor solution, alginic acid sodium salt (alginate) was first dissolved at 3 w% in deionized water (DI). Chitosan (CS) was dissolved at 2 w% in 1% acetic acid solution. For Alg‐Foam hydrogel foam precursor, alginate was further dissolved to a final concentration of 1.5% (w/v). For Alg/CS‐Foam hydrogel foam precursor, CS and alginate solutions were mixed under vigorous stirring to reach final concentration of 1.5 or 2% (w/v) alginate and 0.5 or 1% (w/v) CS. 4 mL of 1 M NaOH solution was added dropwise to 100 mL of Alg/CS‐Foam precursor solution to adjust the pH.

### 4.2 Custom‐Made Fabrication Device

The custom‐made fabrication device was designed in computer assisted design (CAD) and manufactured in‐house using Aluminum EN AW‐6082. The fabrication device consisted of two parts, namely the 382 mL pressure chamber and the lid. A safety valve, a check valve, and an inlet for pressure measurement were embedded in the lid. The nozzle was designed in CAD and fused deposition modeling (FDM) 3d‐printed using an Original Prusa i3 MK3S+ with PLA as the filament.

### 4.3 Hydrogel Foam Fabrication – CaCl_2_ Granules

After filling up the nozzle with 1996.1 ± 12.9 mg of CaCl_2_ granules, the precursor solution was loaded into the chamber of the custom‐made foaming device and tightly closed with the lid. CO_2_ cartridges (Lezyne Inc., USA) were used to inject gas inside of the chamber through the gas injection valve on the lid. CO_2_ was injected up to the desired pressure, followed by manual shaking of the fabrication device to dissolve the gas into the precursor solution. The injection process was repeated until the desired pressure equilibrium was reached. The pressure was measured using a pressure sensor (ABPDANV150PGSA3, Honeywell, Morristown, NJ, USA) connected to the chamber with a tube through a hole in the lid. The amount of injected CO_2_ was calculated following the ideal‐gas equation and Henry's law. For 100 mL of precursor solution, 3.23 gr of CO_2_ was injected in total, resulting in 2.5 gr in the gas phase, and 0.73 gr dissolved in the precursor solution. The extrusion valve was then open while holding the device vertically throughout the extrusion. The remaining calcium granules inside the nozzle were dried in a drying oven and weighed to determine the final concentration of calcium in the hydrogel foam. For experiments where disks were needed, the foam was extruded on a glass slide and immediately covered with a second glass slide with the help of spacers to get a thickness of 0.5 or 1 cm. A biopsy punch with an inner diameter of 0.8 or 1.5 cm was used to make hydrogel foam disks.

### 4.4 Hydrogel Foam Fabrication – CaSO_4_

CaSO_4_ was added into the precursor solution at concentrations from 3.05 mm to 6.1 mm. A static mixer was inserted into the nozzle instead of the CaCl_2_ granules. The hydrogel foam was then fabricated following the same procedure as previously described.

### 4.5 Rheology

Disks of hydrogel foams (0.8 cm in diameter) were used for rheological measurements. Rheological properties of hydrogel foams were characterized using an oscillatory shear rheometer (MCR102, Anton Paar GmbH, Graz, Austria). Measurements were performed at room temperature using an 8 mm parallel plate geometry. Time sweeps (1 Hz, 1% strain) were applied to determine the storage and loss moduli of the hydrogel foams. At least three independent experiments were carried out per experimental group.

### 4.6 Hydrogel Foam Degradation

To measure weight loss over time, Alg‐Foam and Alg/CS‐Foam hydrogel foam disks (1.5 cm in diameter, 1 cm in height) were weighted (*w*_0_) and soaked in 10 mL of simulated uterine fluid (SUF). SUF was prepared following an established protocol [50]. For 1 L of SUF, 4.97 g of NaCl, 0.224 g of KCl, 0.167 g of CaCl_2_, 0.25 g of NaHCO_3_, 0.5 g of glucose, and 0.072 g of NaH_2_PO_4_ dihydrate were dissolved in 1 L of DI. The disks were taken out of the solution after 1, 2, 3, 5, 7, 10, 14, 21, and 28 days, and the wet (*w*_1_) and dry ($w_{1}^{\prime }$) weights were measured. A standard curve for the wet‐to‐dry weight ratio was used to know the initial dry weight ($w_{0}^{\prime }$). The weight loss was calculated as follows:

$$Weight\;loss\;\;\left(\%\right)=\frac{w_{0}^{\prime }-w_{1}^{\prime }}{w_{0}^{\prime }}\;*100\%$$

At least three independent experiments were carried out per experimental group.

### 4.7 Gelation Time

The hydrogel foam was extruded into a centrifuge tube held upside down to show instant gelation. The extrusion was recorded at high‐speed to determine the time of gelation.

### 4.8 Hydrogel Foam Expansion, Physical, and Mechanical Properties

An initial volume of 50 or 100 mL of precursor solution was loaded into the fabrication device and pressurized at 4, 5, or 6 bar. The precursor solution was then extruded into a graduated beaker and the final volume after full expansion was recorded. The expansion rate was defined by the following equation:

$$Expansion\;rate\;\;\left(\%/s\right)=\frac{\left(V_{f}-V_{i}\right)}{\left(t*V_{i}\right)}\;*100\%$$

where *V_i_* and *V_f_* are the initial and final volume of the foam, respectively, and *t* is the time for full expansion. The expansion ratio was calculated as the final hydrogel foam volume over the initial precursor solution volume. The hydrogel foams were then frozen in liquid nitrogen, lyophilized, mounted on samples holders, sputter coated with carbon on a high vacuum sputter (CCU‐010, Safematic, Zizers, Switzerland), and imaged on a scanning electron microscope (SEM) (GeminiSEM 450, ZEISS, Oberkochen, Germany). At least three independent experiments were carried out per experimental group. The wet foam density was determined by measuring the volume (*V*) and wet mass (*w*) of the hydrogel foam. The hydrogel foam was extruded in a centrifuge tube and the resulting hydrogel foam cylinder was cut into disks using a scalpel. The thickness of the disks was measured using a caliper, and the area was measured on pictures of the samples using ImageJ [51]. The wet density of the hydrogel foam was defined as follows:

$$Wet\;density\;\;\left(gr/cm^{3}\right)=\frac{w}{V}$$

The compression strength of the hydrogel foam was tested on a ZwickRoell Z010 (ZwickRoell GmbH & Co. KG, Ulm, Germany). A constant strain rate of 2 mm min^−1^ was applied to the specimen up to 80% strain. The force was measured using a 20 N load cell and the area of the sample was determined as described above.

The dimensional stability of the hydrogel foam was determined in air and in SUF by recording the volume of the hydrogel foam at 0.5, 1, 2, 4 and 6 h and comparing it to the initial volume.

At least three independent experiments were carried out per experimental group.

### 4.9 Blood Coagulation Index

Whole blood coagulation ability of the hydrogel foams was determined by measuring the blood coagulation index. Celox PPH was used a control. Samples were cut into disks (1.5 cm in diameter, 0.5 cm in height) and placed into a 24‐well plate at 37°C for 30 min. Fresh human whole blood from healthy volunteers was collected under the approval of the cantonal ethics commission of the Canton of St. Gallen, Switzerland (BASEC No. 2016‐00816). 1 mL of CPD per 10 mL of whole blood was added to prevent coagulation. The anti‐coagulated whole blood was recalcified by adding 100 µL of 100 mm CaCl_2_ solution to 900 µL of anti‐coagulated whole blood. Directly after mixing, 50 µL of recalcified whole blood was dropped on each disk and incubated at 37°C for 1, 2, 4, 8 or 16 min. After incubation, 2 mL of DI was added gently to each well and incubated for 10 min at 37°C. The supernatant was then measured for absorbance at 540 nm (A_sample_) using a microplate reader (Hidex sense 425‐301, Hidex Oy, Turku, Finland). The absorbance at 540 nm of 50 µL of recalcified whole blood in 2 mL of DI was used as a reference (A_ref_). The BCI was calculated as follows:

$$BCI\;\;\left(\%\right)=\frac{A_{sample}}{A_{ref}}\;*100\%$$

At least three independent experiments were carried out per experimental group.

### 4.10 Free Ca^2+^ Ions

To determine the content of free calcium ions in Alg‐Foam and Alg/CS‐Foam, the hydrogel foams were immersed in 3 mL DI for 6 h. The calcium concentration of the resulting solution was measured using a colorimetric calcium assay (Sigma–Aldrich) according to the manufacturer's instructions and the content of free calcium ions per mass of hydrogel foam was calculated. At least three independent experiments were carried out per experimental group.

### 4.11 RBCs Adhesion

The interactions between the hydrogel foams and red blood cells (RBCs) were quantified by determining the adhesion of RBCs. Celox PPH was used as a control. Samples were cut into disks (1.5 cm in diameter, 0.5 cm in height) and placed into a 24‐well plate at 37°C for 30 min. Fresh human whole blood was obtained from healthy volunteers and collected in EDTA‐coated tubes (Vacutest kima s.r.l., Arzergrande, Italy). The blood was centrifuged at 400 rcf for 15 min and the plasma was discarded to obtain concentrated RBCs. 50 µL of concentrated RBCs were added onto each disk and the samples were incubated at 37°C for 1 h. The samples were then washed with 2 mL PBS and transferred to 4 mL DI. After 1 h of incubation at RT, the absorbance at 540 nm of the supernatant was measured (*A_sample_*). The absorbance at 540 nm of 50 µL of concentrated RBCs in 4 mL DI was used as a reference (*A_ref_*). The proportion of adhered RBCs was calculated using the following equation:

$$Adhered\;RBCs\;\;\left(\%\right)=\frac{A_{sample}}{A_{ref}}\;*100\%$$

At least three independent experiments were carried out per experimental group.

The adhesion of RBCs on the different samples was observed using SEM. The disks of hydrogel foams or Celox PPH were placed in a 24‐well plate and 50 µL of concentrated RBCs were dropped onto each sample followed by incubation at 37°C for 1 h. The samples were then rinsed with PBS and fixed with 3% glutaraldehyde overnight. The samples were dried by immersion in 50, 70, 90% EtOH for 15 min for each EtOH concentration. The samples were then immersed three times 15 min in 100% EtOH, mounted on SEM sample holders, sputter coated with carbon, and imaged on a SEM.

### 4.12 Hemolysis

The hemolytic activity of hydrogel foams was determined by measuring the release of hemoglobin. Celox PPH was used as a control. Samples were cut into disks (1.5 cm in diameter, 0.5 cm in height) and placed into 15 mL centrifuge tubes at 37°C for 30 min. 5 mL of normal saline (0.9% NaCl) was added to each tube and incubated at 37°C for 30 min. Diluted blood was prepared by mixing concentrated RBCs and normal saline at a 2:3 volume ratio. 0.1 mL of diluted blood was added to each tube, followed by incubation at 37°C for 1 h. 1 mL of the solution in each tube was then collected and centrifuged at 800 rcf for 5 min. The absorbance of the resulting supernatant was measured at 540 nm for each sample (*A_sample_*). The absorbance of 0.1 mL of diluted blood in 5 mL normal saline or 5 mL of DI served as a negative (*A_neg_*) and positive (*A_pos_*) control, respectively. The hemolysis ratio was calculated using the following equation:

$$Hemolysis\;\;\left(\%\right)=\frac{A_{sample}-A_{neg}}{A_{pos}-A_{neg}}\;*100\%$$

At least three independent experiments were carried out per experimental group.

### 4.13 Biocompatibility of Hydrogel Foam

Biocompatibility of the hydrogel foams was determined through perfused cell medium experiments adapted from a reported procedure [52]. Endometriotic cells were cultured under standard culture conditions at 37°C with 5% CO_2_ using Dulbecco's Modified Eagle's Medium (DMEM) high glucose, with 10% Fetal calf serum, 1% L‐Glutamine, 1% Penicillin‐Streptomycin‐Neomycin as full growth medium. 4000 endometriotic cells were seeded in 100 µL full growth medium in a 96‐well plate and incubated for at least 24 h under standard culture conditions. Hydrogel foams and Celox PPH were cut into disks (1.5 cm in diameter, 1 cm in height) and incubated in 3 mL of full growth medium at 37°C for 24 h to get infused medium. Medium was removed from cells and 50 µL of fresh media and 50 µL of infused media was added to the cells. Also, 100 µL of fresh full growth medium were added to the cells as control. The plate was then incubated under standard culture conditions for 24 h. Cell viability was assessed through the ATP dependent CellTiter‐Glo 2.0 Cell Viability Assay (#G9241, Promega, Dübendorf, Switzerland) and cytotoxicity was measured using the LDH dependent CytoTox 96 Cytotoxicity assay (#G1780, Promega, Dübendorf, Switzerland) according to the manufacturer's instructions. In the cytotoxicity assay, 10 µL of 10X Lysis Solution was added to cells as a positive control and the absorbance was recorded after exactly 30 min and the stop solution was not used. At least four independent experiments were carried out per experimental group.

### 4.14 Hysterectomy Biopsy

Potential tissue damage was investigated by contacting the hydrogel foams on fresh uterine wall biopsies after hysterectomy collected from healthy donors using an 8 mm biopsy punch. Celox PPH was used as a control. Hydrogel foam and Celox PPH were cut into disks of 1.5 cm in diameter and 0.5 cm in height and placed into a 12‐well plate. The biopsies were placed on top of the samples to contact the endometrium and the tested materials. The wells were filled with PBS to avoid drying. The samples were incubated at 37°C for 24 h and subsequently, the biopsies were collected and sent to Sophistolab AG where they were block paraffinized, cut and stained using H&E. Histology scans were taken on an Aperio GT 450 DX (Leica Biosystems, Nussloch, Germany) and analyzed using ImageScope.

### 4.15 Antibacterial Activity

Antibacterial activity was assessed using previously reported zone inhibition assay [53]. Briefly, *Staphylococcus Aureus* (*S. aureus*) and *Escherichia Coli* (*E. coli*) were cultivated overnight in Brain‐Heart Infusion (BHI) growth medium. Cefuroxime (CFX) was added to the hydrogel foam precursor solution at a final concentration of 1%. Hydrogel foams were then fabricated following the usual protocol, cut into disks (1.5 cm diameter, 0.8 cm height), and sterilized under UV light for 30 min. 200 µL of overnight E. coli and S. aureus cultures at 10^8^ CFU mL^−1^ were spread evenly on an agar plate supplemented with BHI. The sterilized hydrogel foam disks were placed on the agar plates. Alg/CS‐Foam without CFX and a sterile paper previously soaked in 1% CFX solution were used as controls. The agar plates were then incubated at 37°C for 24 h. The area of inhibition around each sample was measured from images of the agar plates using ImageJ. At least five independent experiments were carried out per experimental group.

### 4.16 Hemostatic Drug Release

The release profile of tranexamic acid (TXA) was assessed using fluorescamine derivatization based on an existing protocol [54]. The hydrogel foam was prepared as described before, except that the alginate was dissolved in 0.4% (w/v) TXA in DI. The hydrogel foam was cut into disks (1.5 cm in diameter, 0.5 cm in height) and weighed to calculate the initial mass of TXA. Samples were then put in a 6‐well plate and immersed in 10 mL PBS. After 0.25, 0.5, 1, 2, 3, and 5 h, 12 µL of solution was collected, diluted 10x in DI, and mixed with 240 µL of fluorescamine. After 1 h incubation in the dark at RT, the fluorescence at 355 nm (BW40) was measured on a Hidex plate reader. The linearity of the response was verified over the concentration range 1.5625–50 µg mL^−1^ (R^2^≥0.9998). At least three independent experiments were carried out.

### 4.17 Accelerated Degradation

The degradation solution for accelerated degradation was evaluated by measuring the weight loss of the hydrogel foams over time. The accelerated degradation solution (ADS) consisted in 0.9% NaCl and 0.15 mg mL^−1^ alginate lyase. Normal saline and SUF were used for comparison. Samples were cut into disks of 1.5 cm (large) or 0.8 mm (small) in diameter and 1 cm in height, weighted (w_0_), and placed in 6‐ or 24‐well plates, respectively. Large and small samples were immersed respectively in 10 mL or 2.85 mL of ADS, Saline, or SUF for 6 h at 37°C. The immersion solutions were changed every 2 h. To calculate the weight loss, the large samples were taken out and processed as previously described in the 28 days degradation to obtain the initial ($w_{0}^{\prime }$) and the final ($w_{1}^{\prime }$) dry weights. To investigate changes in rheological properties, the small samples were characterized using an oscillatory shear rheometer as described above. At least three independent experiments were carried out per experimental group.

### 4.18 Biocompatibility of Degradation Products

Biocompatibility of the accelerated degradation solution and degradation products of the hydrogel foams was determined for normal human dermal fibroblasts (NHDFs). NHDFs were cultured under standard culture conditions at 37°C with 5% CO_2_ using Dulbecco's Modified Eagle's Medium (DMEM) high glucose, with 10% Fetal calf serum, 1% Glutamax, 1% Penicillin‐Streptomycin‐Neomycin as full growth medium. 4000 NHDFs were seeded in 100 µL full growth medium in a 96‐well plate and incubated for at least 24 h under standard culture conditions. Alg‐Foam and Alg/CS‐Foam were incubated in 3 mL of 0.9% NaCl or ADS at 37°C for 6 h to get infused solutions. Medium was removed from cells and 50 µL of fresh media and 50 µL of infused solution was added to the cells. 100 µL of fresh full growth medium or 50 µL of fresh media and 50 µL of 0.9% NaCl were added to the cells as controls. The plate was then incubated under standard culture conditions for 24 h. Cell viability was assessed through the ATP dependent CellTiter‐Glo 2.0 Cell Viability Assay (#G9241, Promega, Dübendorf, Switzerland) and cytotoxicity was measured using the LDH dependent CytoTox 96 Cytotoxicity assay (#G1780, Promega, Dübendorf, Switzerland) according to the manufacturer's instructions. In the cytotoxicity assay, 10 µL of 10X Lysis Solution was added to cells as a positive control and the absorbance was recorded after exactly 30 min and the stop solution was not used. At least four independent experiments were carried out per experimental group.

### 4.19 pH, Osmolality, and Residual Acetic Acid

To determine the pH and osmolality of the material, Alg‐Foam and Alg/CS‐Foam were cut into disks (1.5 cm in diameter, 1 cm in height) and immersed in 8 mL DI for 6 h. The pH of the resulting solution was measured after 0.5, 1, 2, 4, and 6 h. Osmolality of the solution was measured after 6 h immersion and the osmolality of the foam was calculated as follows:

$$Osmolality\;\;\left(mOsm/kg\right)=\frac{osmo_{solution}*V_{solution}}{w_{sample}}$$

where *osmo_solution_* is the osmolality of the solution, *V_solution_* is the volume of the solution, and *w_sample_* is the weight of the hydrogel foam sample. At least three independent experiments were carried out per experimental group. To measure the residual acetic acid, Alg/CS‐Foam was immersed overnight in DMSO containing 0.3 wt.% maleic acid (MA) and in D_2_O containing 0.5 wt.% potassium hydrogen phthalate (KHP). The ^1^H‐NMR spectrum of the resulting solutions was obtained on a 400 MHz NMR spectrometer (Bruker Avance III 400, Bruker, Billerica, Massachusetts, U.S.). The acetic acid content was quantified by comparing the peak integrals of acetic acid to those of the reference compounds.

### 4.20 Balloon Model

To simulate the atonic uterus, an in vitro model using layered party balloons was created. One standard party balloon was inserted into a second party balloon. The tip of the balloons simulated the fundus of the uterus, and the neck simulated the cervix. A hole was cut through the tip of both balloons to place a 3D printed conical connector secured to the model with parafilm and linked to a pressure sensor (ABPDANV015PGSA3, Honeywell) by a polyethylene (PE) tube to measure the pressure at the simulated fundus. Another PE tube was placed through the neck of the balloons and connected to a second pressure sensor to record the pressure from the simulated cervix. An additional part was added on the nozzle to hold the tube. Pressure measurements were recorded using computer software PuTTY [55]. To mimic the uterine environment, the model was fully immersed in SUF before, during, and after the extrusion.

### 4.21 Simulation

A two‐way fluid–structure interaction (FSI) simulation was conducted in ANSYS Workbench 2024 R2 [56] to model the mechanical response of the uterus to hydrogel precursor expansion following an adapted procedure [29]. A rotationally symmetric 3D geometry was created. The model comprised a fluid domain (CO_2_‐filled cavity) and a solid domain (uterine wall, thickness 4 mm), with an initial cavity volume of 538 mL. The uterine wall was modelled using a two‐parameter incompressible Mooney‐Rivlin material (C_10_  =  60 000 Pa, C_01_  =  12 000 Pa, ρ  =  1048 kg/m^3^, D1 =  1 × 10^−6^ Pa^−1^). Structural boundary conditions fixed the fundus in radial direction (X and Z) while allowing axial (Y) displacement; the cervical opening was fully constrained. A time‐dependent outward pressure load, P(t) = 950·(t/15)^2^ Pa, simulated precursor expansion. In the fluid domain, laminar CO_2_ flow was modelled with a mass flow inlet of 6.7 × 10^−5^ kg/s. The inner cavity wall was set as an FSI interface with dynamic mesh enabled. System Coupling facilitated synchronized data exchange between Fluent and Transient Structural at each 0.2 s time step over a total duration of 15 s.

### 4.22 Ex Vivo Bleeding Model with Human Placenta

To simulate the diffuse PPH bleeding from the uterine wall, a fresh human placenta was perfused with fresh human blood and placed in a 3D printed uterus model adapted from the model developed by Candidori et al. [57]. Human placentas and blood were obtained from uncomplicated term pregnancies after caesarean section from the Kantonsspital St Gallen (KSSG). The project was approved by the local ethics committee (EKSG 10/078, BASEC No. 2026‐01218) and performed in accordance with the principles of the Declaration of Helsinki. Written informed consent was obtained prior to delivery. The umbilical cord was cut at ∼5 cm above the placental insertion and inserted through a hole at the tip of the uterus model. A PE tube for blood perfusion was inserted inside of the umbilical artery and secured with plastic forceps and a rubber band. The placenta was scraped with a surgical scalpel to allow for diffuse bleeding. The blood was then perfused at a rate of approximately 2.5 mL sec^−1^ and the hydrogel foam was injected inside of the cavity by placing the nozzle through the neck of the model. Once the injection was completed, the model was open and the hydrogel foam was evaluated for adequate crosslinking.

### 4.23 Accelerated Degradation in Balloon Model

The accelerated degradation of Alg/CS‐Foam was tested in the uterus balloon model. Hydrogel foam was extruded into the balloon model, weighed, and immersed for 6 h in SUF. Subsequently, the model were removed from SUF, remaining SUF was poured out of the model, and ADS was injected inside of the model. A model injected with SUF served as control. After 6 h, ADS was poured out and the models were weighed again. The weight loss was measured as previously described. At least three independent experiments were carried out per experimental group.

### 4.24 Ex Vivo Swine Uterus Model

Fresh swine uteri were collected from a local slaughterhouse. Each uterus was prepared by carefully isolating the vaginal canal and the uterine horns, with surrounding connective tissue removed using surgical scissors. Throughout the preparation and experimentation, PBS was regularly applied to prevent tissue dehydration. A PE tube connected to a pressure sensor was secured inside one of the horn while the other horn was sealed with a plastic clamp. The hydrogel foam was injected thought the vaginal canal using the nozzle described for the balloon model, with the PE tube for pressure measurement and with the condom for adequate sealing.

After completing hydrogel foam extrusion and pressure measurement in the ex vivo porcine uterus, ultrasound imaging was performed with a Clarius L7 HD linear wireless probe (Clarius, Vancouver, Canada) to visualize the distribution of the injected hydrogel foam within the uterine cavity. An agar ultrasound phantom was placed directly above the tissue sample.

### 4.25 Animal Ethics and Experimental Design

All experimental procedures involving pigs were conducted according to a protocol approved by the Commission on Work with Experimental Animals at the Faculty of Medicine in Pilsen, Charles University, and supervised by the Ministry of Education, Youth and Sports of the Czech Republic (project code: MSMT‐25292/2025‐4). All procedures complied with the applicable laws of the Czech Republic and the European Union directive on the protection of animals used for scientific purposes.

### 4.26 Anesthesia and Preoperative Preparation

Upon arrival at the facility, all animals underwent a quarantine period and clinical examination by the attending veterinarian. Prior to surgery, each animal was weighed to allow for accurate dosing of anesthetics. General anesthesia was induced via intramuscular administration of ketamine (10 mg kg^−1^; Narkamon 100 mg mL^−1^, BioVeta a.s., Ivanovice na Hané, Czech Republic), azaperone (4 mg kg^−1^; Stresnil 40 mg mL^−1^, Elanco AH, Prague, Czech Republic), and atropine (1 mg; Atropin Biotika, Hoechst Biotika, Martin, Slovak Republic). Anesthesia was maintained with an intravenous bolus of propofol (2.5 mg kg^−1^; Propofol 2% MCT/LCT, Fresenius Medical Care a.s.), followed by endotracheal intubation using a laryngeal tube. Continuous anesthesia was ensured through an intravenous infusion of propofol (6–10 mg/kg/h) combined with nalbuphine (0.5 mg/kg/h; Nalbuphin, Torrex Chiesi CZ s.r.o., Prague, Czech Republic) to provide adequate analgesia during the procedure.

### 4.27 In Vivo Uterus Injection, Ultrasound Imaging, and Tissue Sampling

A healthy female Prestice black‐pied pig (35 kg, 5 months old) was used for the uterine injection experiment. Following midline laparotomy, the left uterine horn was ligated to serve as a native tissue control. A 2‐cm incision was made above the cervix to enable insertion of the injection nozzle. Hydrogel foam was injected into the uterine lumen while intrauterine pressure was continuously monitored via a tube connected to a pressure sensor.

Ultrasound imaging (Clarius linear probe) was performed before, during, and after the injection to visualize foam propagation within the uterus. Upon completion, the uterus was excised for histological evaluation of potential tissue injury. Both the control and treated horns were longitudinally sectioned and sent to Sophistolab AG (Germany), where they were paraffin‐embedded, sectioned, and stained with hematoxylin and eosin (H&E). Whole‐slide imaging was performed using an Aperio GT450 DX scanner, and the samples were analyzed using ImageScope software. After collection of all histological samples, the animal was humanely euthanized.

### 4.28 Liver Defect Model and Hemostatic Evaluation

3 healthy Prestice black‐pied pigs (32 to 49 kg, 5 months old) were used for the liver defect experiment with a sex ratio of 1:2 male to female. The in vivo hemostatic performance of the hydrogel foam was evaluated in a standardized columniform liver defect model, with Celox PPH serving as a reference control. Approximately 100 µL of pre‐prepared hydrogel foam or Celox PPH were loaded into 1‐mL syringes (Injekt‐F, B. Braun, Melsungen, Germany). Both materials were also cut into cylindrical disks (1.5 cm diameter, 0.5 cm height). A superficial liver defect (5 mm in diameter) was created parallel to the organ surface using a biopsy punch and surgical scissors. After removal of excess blood, the respective hemostatic material (hydrogel foam or Celox PPH) was applied into the defect, and the disk was placed on top. Gentle manual compression was maintained for 1.5 min, after which remaining blood loss was collected using pre‐weighed gauze. Untreated defects managed by compression alone served as negative controls. If hemostasis was not achieved, blood loss was measured for 2 min following compression removal, and the defect was subsequently cauterized. Following collection of histological samples, the animal was humanely euthanized.

### 4.29 Statistical Analysis

Data are presented as mean ± standard deviation. Significance and *p* values were determined using ANOVA test followed by Tukey's HSD test. NS *p*>0.05, * *p*<0.05, ** *p*<0.01, *** *p*<0.001. RStudio was used to perform the statistical analyses [58].

## Author Contributions

**Charlotte Meyer**: conceptualization, methodology, data curation, investigation, validation, formal analysis, visualization, project administration, writing – original draft, writing – review and editing, software. **Noël Strasser**: investigation, writing – review and editing, visualization. **Ilakkiya Ravindrarajah**: investigation, writing – review and editing. **Elsa Wrenger**: investigation, writing – review and editing. **Kristian Ivkovic**: investigation, writing – review and editing. **Jan Ševčík**: investigation, writing – review and editing. **Sima Šarčević**: investigation, writing – review and editing. **Lenka Červenková**: writing – review and editing, investigation. **Jáchym Rosendorf**: writing – review and editing, supervision. **Václav Liška**: supervision, writing – review and editing. **Alexandre H. Anthis**: writing – review and editing, conceptualization. **Thomas Rduch**: conceptualization, methodology, writing – review and editing. **Inge K. Herrmann**: conceptualization, validation, supervision, funding acquisition, project administration, resources, writing – review and editing, writing – original draft.

## Conflicts of Interest

C.M., T.R. and I.K.H. declare that a patent application has been filed related to the material foaming and deployment technology reported in this publication. The patents have been filed by Empa, ETH Zurich, and the University of Zürich (Charlotte Meyer, Thomas Rduch, Inge K. Herrmann, EP26184374.2, patent filed). All other authors report no conflicts of interest.

## Supporting information




**Supporting File 1**: advs77669‐sup‐0001‐SuppMat.docx.

**Supporting File 2**: advs77669‐sup‐0002‐VideoS1.mov.

**Supporting File 3**: advs77669‐sup‐0003‐VideoS2.mov.

**Supporting File 4**: advs77669‐sup‐0004‐VideoS3.mov.

## Data Availability

The data that support the findings of this study are available from the corresponding author upon reasonable request.
