# Supplementary material for: An Injectable, Self‐Expanding Hemostatic Foam for Field‐Compatible Postpartum Hemorrhage Management
Source: Adv Sci (Weinh). 2026 Sep 27:e77669. Online ahead of print. doi: 10.1002/advs.77669 (PMC13616286; doi:10.1002/advs.77669)
Supplement: Supplementary file 1 [file ADVS-9999-e77669-s003.docx]

An Injectable, Self-Expanding Hemostatic Foam for Field-Compatible Postpartum Hemorrhage Management

*Charlotte Meyer,^1-4^ Noël Strasser,^2^ Ilakkiya Ravindrarajah,^1,2^ Elsa Wrenger,^1,2^ Kristian Ivkovic,^1,2^ Jan Ševčík,^5,6^ Sima Šarčević,^5,6^ Lenka Červenková,^6^ Jáchym Rosendorf,^5,6^ Václav Liška,^5,6^ Alexandre H. Anthis,^1-4^ Thomas Rduch,^3,7^ Inge K. Herrmann^1-4^**

^1^ The Ingenuity Lab, Balgrist University Hospital, Forchstrasse 340, 8008 Zurich, Switzerland.

^2^ Nanoparticle Systems Engineering Laboratory, Institute of Energy and Process Engineering (IEPE), Department of Mechanical and Process Engineering (D-MAVT), ETH Zurich, Sonneggstrasse 3, 8092 Zurich, Switzerland.

^3^ Nanomaterials in Health Laboratory, Department of Materials Meet Life, Swiss Federal Laboratories for Materials Science and Technology (Empa), Lerchenfeldstrasse 5, 9014 St. Gallen, Switzerland.

^4^ Faculty of Medicine, University of Zurich, Rämistrasse 71, 8006 Zurich, Switzerland.

^5^ Department of Surgery, Faculty of Medicine in Pilsen, Charles University, Prague, Czech Republic.

^6^ Biomedical Center, Faculty of Medicine in Pilsen, Charles University, Prague, Czech Republic.

^7^ Department of Gynecology, Cantonal Hospital St Gallen (KSSG), Rorschacherstrasse 95, CH-9007 St Gallen, Switzerland.

[*ingeh@ethz.ch](mailto:*ingeh@ethz.ch), [inge.herrmann@uzh.ch](mailto:inge.herrmann@uzh.ch), [inge.herrmann@empa.ch](mailto:inge.herrmann@empa.ch), +41 (0)58 765 7153

# SUPPORTING INFORMATION

Supplementary Tables

Table S1. Details of batch reproducibility, storage stability, and success rate of Alg/CS-Foam fabrication by trained (1) and untrained (2-4) operators with two different lots of each polymer.

| Operator n° | Alginate lot n° | Chitosan lot n° | Storage | Success |
| --- | --- | --- | --- | --- |
| 1 | MKCW2126 | BCCM085 | Original package | Yes |
| 2 | MKCW2126 | BCCM0851 | Original package | Yes |
| 3 | MKCW2126 | BCCM0851 | Original package | No, technical failure |
| 4 | MKCW2126 | BCCM0851 | Original package | Yes |
| 1 | MKCW2126 | BCCM0851 | Original package | No, expansion ratio too low |
| 1 | MKCS7881 | BCCG5629 | Original package | Yes |
| 1 | MKCS7881 | BCCG5629 | Original package | Yes |
| 1 | MKCS7881 | BCCG5629 | Original package | Yes |
| 1 | MKCS7881 | BCCM0851 | Original package | Yes |
| 1 | MKCS7881 | BCCG5629 | In Eppendorf tube for 1.5 year | Yes |

Supplementary Figures

Figure S1: Technical drawing of fabrication device composed of the lid, an O-ring, and the chamber.

Figure S2: Macroscopic image of fabrication device.

Figure S3: Schemes of 3D printed nozzles. Nozzle filled with CaCl_2_ granules retained by a mesh (left) and nozzle combined with static mixer for CaSO_4_ dissolution (right).

Figure S4: Volumes of initial precursor solution before extrusion (InitialFoam), crosslinked hydrogel foam (Foam@2min) and remaining precursor solution (Liquid@2min) 2 min after extrusion when using 1.5 or 2% alginate and 0.5 or 1% chitosan in the precursor solution. Data are presented as mean ± standard deviation (n=3).

Figure S5: Ca^2+^ concentration in hydrogel foam made with different initial precursor solution volumes. Data are presented as mean ± standard deviation (n=3).

Figure S6: Representative rheological measurements of Alg-Foam and Alg/CS-Foam.

Figure S7: *In vitro* degradation of Alg-Foam and Alg/CS-Foam in simulated uterine fluid over 28 days.

Figure S8: Scheme of reconstitution and application workflow with time for each step (n=4).

Figure S9: Osmolality of Alg-Foam and Alg/CS-Foam. Data are presented as mean ± standard deviation (n=3).

Figure S10: Cell viability (left) and cytotoxicity (right) of degradation products of Alg/CS-Foam after immersion in saline and ADS. Cell medium was used as control for cell viability. Saline and lysed cells were used as controls for cytotoxicity. Data are presented as mean ± standard deviation (n=4). Significance and P values were determined using ANOVA test followed by Tukey’s HSD test. NS P>0.05, * P<0.05, ** P<0.01, *** P<0.001.

Figure S11: pH values of Alg-Foam and Alg/CS-Foam over 6 hours.

Figure S12: ^1^H-NMR spectrum of residual acetic acid in DMSO containing 0.3 wt% maleic acid (MA) (left) and in D_2_O containing 0.5 wt% potassium hydrogen phthalate (KHP) (right).

Figure S13: *In vitro* intrauterine pressure during repeated hydrogel foam injection. Green arrows indicate the three consecutive injections.

Figure S14: *In vitro* intrauterine pressure measured from the cervix during hydrogel foam injection in the uterine model immersed in SUF.

Figure S15: *In vitro* wet weight loss of Alg/CS-Foam when exposed for 6 hours to SUF and ADS inside of the uterine model. Data are presented as mean ± standard deviation (n=3).

Figure S16: Macroscopic images of the hemostatic effect of Alg/CS-Foam+TXA. Dotted circles outline the defect boundaries.

Figure S17: *In vivo* blood loss after application of Alg/CS-Foam, Alg/CS-Foam+TXA, and Celox™ compared to no material accounting for biological variability. Data are presented as mean ± standard deviation (n=3).
